# Oxidovanadium(V) Schiff Base Complexes Derived from Chiral 3-amino-1,2-propanediol Enantiomers: Synthesis, Spectroscopic Studies, Catalytic and Biological Activity

**DOI:** 10.3390/ijms25095010

**Published:** 2024-05-03

**Authors:** Grzegorz Romanowski, Justyna Budka, Iwona Inkielewicz-Stepniak

**Affiliations:** 1 Faculty of Chemistry, University of Gdansk, Wita Stwosza 63, PL-80308 Gdansk, Poland; 2 Department of Pharmaceutical Pathophysiology, Faculty of Pharmacy, Medical University of Gdansk, Dębinki 7, Building 27, PL-80211 Gdansk, Poland; justyna.budka@gumed.edu.pl

**Keywords:** vanadium, epoxidation, cytotoxicity, cytoprotection

## Abstract

Oxidovanadium(V) complexes, **[(+)VOL^1-5^]** and **[(–)VOL^1-5^]**, with chiral tetradentate Schiff bases, which are products of monocondensation of *S*(‒)-3-amino-1,2-propanediol or *R*(+)-3-amino-1,2-propanediol with salicylaldehyde derivatives, have been synthesized. Different spectroscopic methods, viz. ^1^H and ^51^V NMR, IR, UV-Vis, and circular dichroism, as well as elemental analysis, have been used for their detailed characterization. Furthermore, the epoxidation of styrene, cyclohexene, and two monoterpenes, *S*(‒)-limonene and (‒)-α-pinene, using two oxidants, aqueous 30% H_2_O_2_ or *tert*-butyl hydroperoxide (TBHP) in decane, has been studied with catalytic amounts of all complexes. Finally, biological cytotoxicity studies have also been performed with these oxidovanadium(V) compounds for comparison with *cis*-dioxidomolybdenum(VI) Schiff base complexes with the same chiral ligands, as well as to determine the cytoprotection against the oxidative damage caused by 30% H_2_O_2_ in the HT-22 hippocampal neuronal cells in the range of their 10–100 μM concentration.

## 1. Introduction

Vanadium is an abundant element in a number of various biological systems [1]. Many examples of vanadium enzymes and compounds can be found in nature. Among them are vanadium nitrogenases found in bacteria [2,3], vanadium-dependent haloperoxidases in terrestrial fungi and lichen or marine algae [4,5,6], or natural compound amavadin found in the mushroom *Amanita muscaria* [7]. Catalytic activity studies of vanadium complexes have been stimulated recently by their importance in modeling the active centers of such enzymes in many biological processes [4,8] and towards diverse organic transformations [9,10]. Salicylaldimine-derived chiral Schiff bases very easily coordinate with vanadium ions and exhibit widespread applications [11,12]. Among them, chiral Schiff bases derived from *N*-salicyl-β-amino alcohols are considered “privileged ligands” with fine-tuned structural and electronic properties [13,14]. Multidentate unsymmetrical vanadium(V) Schiff base complexes with such chiral ligands have been widely used as catalysts in the asymmetric alkynylation of aldehydes [15], enantioselective oxidation of organic sulfides [15,16,17], stereoselective synthesis of tetrahydrofurans [18,19], epoxidation of alkenes [20,21], and oxidation of bromide [18]. It is noteworthy that nowadays, epoxidation of various olefins is a widely studied reaction in organic chemistry since epoxides have commonly been used as important intermediates for the synthesis of a wide range of commercial products, as well as useful pharmaceuticals in medicine [22]. Moreover, the formation of new carbon–carbon bonds by ring-opening reactions make optically pure epoxides especially valuable key intermediates and products in different areas of organic chemistry [23].

Vanadium complexes have extensively been reported as prospective medicinal pharmaceutics [24], mentioning their wide biological activities. Lately, their therapeutic potential against pneumonia [25] and tuberculosis bacteria [26] has been found, as well as SARS-CoV-2 [27] and HIV [28] viruses. Moreover, the interest of scientists has lately been increasingly observed in studies on various vanadium complexes possessing anticancer [29], antiparasitic [30], anti-inflammatory [31], or antidiabetic [32,33] properties, an ability that is also cytoprotective against the oxidative damage made by reactive oxygen species to hippocampal cells [34,35]. In various neurodegenerative diseases, such as stroke and brain trauma but also Alzheimer’s and Parkinson’s diseases, oxidative stress is a very consistent component. An excellent model cell for developing new innovative cytoprotective drugs and studying the consequences of endogenous oxidative stress is the hippocampal cell line HT-22 [36]. 

**Figure 1:**
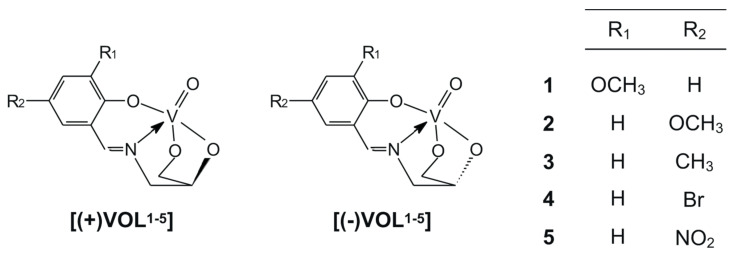
Structural formulae of oxidovanadium(V) complexes.

New oxidovanadium(V) complexes with chiral tetradentate Schiff base, products of monocondensation reaction of substituted salicylaldehyde derivatives with chiral amino alcohols, i.e. *R*(+)-3-amino-1,2-propanediol and *S*(‒)-3-amino-1,2-propanediol (Figure 1) are described within this paper. Their structures were confirmed by spectroscopic techniques, i.e., ^1^H and ^51^V NMR, IR, UV-Vis, and circular dichroism, as well as elemental analysis. Furthermore, their catalytic activity has been studied in the epoxidation of alkenes, i.e., styrene, cyclohexene, and naturally occurring monoterpenes, i.e., *S*(−)-limonene and (−)-α-pinene, during the oxidation reaction by 30% H_2_O_2_ or *tert*-butyl hydroperoxide. Biological cytotoxicity studies have also been performed with these oxidovanadium(V) compounds and for comparison with *cis*-dioxidomolybdenum(VI) complexes with the same chiral ligands. Finally, their cytoprotective properties have also been studied against the oxidative damage caused by 30% H_2_O_2_ in the HT-22 hippocampal neuronal cells in the range of their 10–100 μM concentration.

## 2. Results and Discussion

### 2.1. Infrared Spectra

In IR spectra of all chiral oxidovanadium(V) complexes, **[(+)VOL^1-5^]** and **[(−)VOL^1-5^]**, strong and sharp bands appear in the 956–998 cm^−1^ region, which is characteristic of single V=O stretching modes. They are similar to the values found for the other oxidovanadium(V) Schiff base complexes [37]. Furthermore, characteristic imine C=N stretching vibrations at 1640–1652 cm^−1^ indicate the coordination of chiral Schiff base ligands to the vanadium(V) ion [38]. The deprotonation of all coordinated hydroxyl (phenolic and alcoholic) groups of Schiff base ligands is proved by the lack of any absorption bands in the range of 3200–3700 cm^−1^. Moreover, the coordination of all these phenolate and alkoxide oxygens to vanadium atoms is shown by strong asymmetric and symmetric ν(C-O) vibrations at 1277–1316 and 1046–1095 cm^−1^, respectively (Figure S1). In the case of **[(+)VOL^5^]** and **[(−)VOL^5^]**, with nitro substituent in salicylaldimine fragment, asymmetric and symmetric ν(NO_2_) vibrations have been noticed at ca. 1560 and 1340 cm^−1^, respectively.

### 2.2. UV-Vis and Circular Dichroism Spectra

UV-Vis spectra of all oxidovanadium(V) complexes show the low-energy absorption bands belonging to electron LMCT transitions (Figure S2) from phenolate oxygen p_π_ orbital to an empty d orbital of vanadium atom, appearing between 339 and 359 nm [39]. The circular dichroism spectra have also revealed two bands from intraligand π‒π* transitions in 278–281, 304–322, and additional bands in the 362–390 nm regions of the same origin as it was attributed for UV-Vis spectra. Moreover, in the case of **[(+)VOL^1-5^]**, the latter π‒π* transitions and LMCT transitions have the same positive Cotton effects in contrast to **[(−)VOL^1-5^]** with negative Cotton effects for these bands. Finally, it is noteworthy that all absorption bands exhibit exactly opposite Cotton effects when comparing **[(+)VOL^1-5^]** and **[(−)VOL^1-5^]** compounds, which possess asymmetric carbons in amino alcohol moiety with *R* and *S* configuration, respectively (Figure S3).

### 2.3. NMR Measurements

For characterization of all oxidovanadium(V) Schiff base complexes, ^1^H and ^51^V NMR spectra were recorded in DMSO-d_6_. Schiff bases complexes derived from *S*(‒)-3-amino-1,2-propanediol and *R*(+)-3-amino-1,2-propanediol show identical spectra; therefore, only the latter compounds have been measured. Successful condensation of all salicylaldehyde derivatives with each chiral amino alcohol has been proved by the presence of azomethine proton signals in the case of all complexes. The two-dimensional NMR experiments (COSY and NOESY) were performed to establish proximity and connection between all protons but also for the assignment and identification of all proton signals in the case of the **[(+)VOL^4^]** compound. ^1^H spectrum, with the help of the COSY experiment (Figure S4), unambiguously reveal a connection between methine proton (signal at 5.19 ppm) with all four methylene protons doublets of doublets. Furthermore, for the signals between two methylene protons neighboring an oxygen atom (4.89 and 5.44 ppm), as well as two methylene protons attached to a nitrogen atom (3.75 and 4.74 ppm), the cross-peaks have been found. Moreover, a cross-peak between two aromatic protons (6.71 and 7.52 ppm) has been found showing their close neighborhood. On the other hand, the information regarding the close spatial proximity between the 4.74 ppm methylene doublet of doublets and azomethine proton signal at 8.70 ppm is given by a cross-peak in NOESY spectrum [40], as well as another cross-peak between azomethine proton signal and one of the aromatic proton signal at 7.71 was useful to assign aromatic ring protons signals. During the ^51^V NMR experiments single signals in the range of -529.8 to -532.9 ppm have been noticed, indicating the presence of oxidovanadium(V) complexes only in the monomeric forms.

### 2.4. Catalytic Oxidation Studies

**Figure 2:**
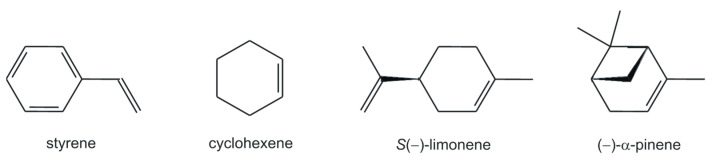
Olefinic and monoterpene substrates chosen for catalytic studies.

Oxidovanadium(V) complexes obtained in this paper, i.e., **[(+)VOL^1-5^]** and **[(−)VOL^1-5^]**, have been checked as catalysts in the oxidation of alkenes, such as styrene, cyclohexene, and additionally, naturally occurring monoterpenes, i.e., *S*(−)-limonene and (−)-α-pinene (Figure 2). As the best solvent for these oxidation reactions, 1,2-dichloroethane (DCE) was chosen with *tert*-butyl hydroperoxide (TBHP) as the terminal oxidant, compared to the other common solvents like acetonitrile, toluene, ethanol, methanol, chloroform, and methylene chloride. According to our observation, for reflux conditions, the lower reaction temperature of the latter solvents is responsible for the poorer conversions of the substrates. On the other hand, when 30% H_2_O_2_ was employed to avoid a biphasic system, acetonitrile was chosen as the best solvent. The oxidation reactions with all substrates needed 5 h to reach completion, and to achieve the best conversions and selectivities, the reactions required solvents with higher reaction temperatures for their reflux conditions, as it was similarly concluded previously [41]. The effect of various reaction parameters was taken into account to achieve appropriate reaction conditions for the best conversions. For this purpose, each of the oxidants’ molar ratios to the substrate has been compared, i.e., 1:1, 2:1, 3:1, and 4:1, as well as the different amounts of catalyst used, i.e., 0.5, 1, 2, and 3 mol%, during our test reactions. Our observations have led to the conclusion that the 3:1 molar ratio of oxidant to each substrate is optimal, and 1 mol% loading of catalyst is a sufficient amount for running the oxidations in optimized reaction conditions.

**Figure 3:**
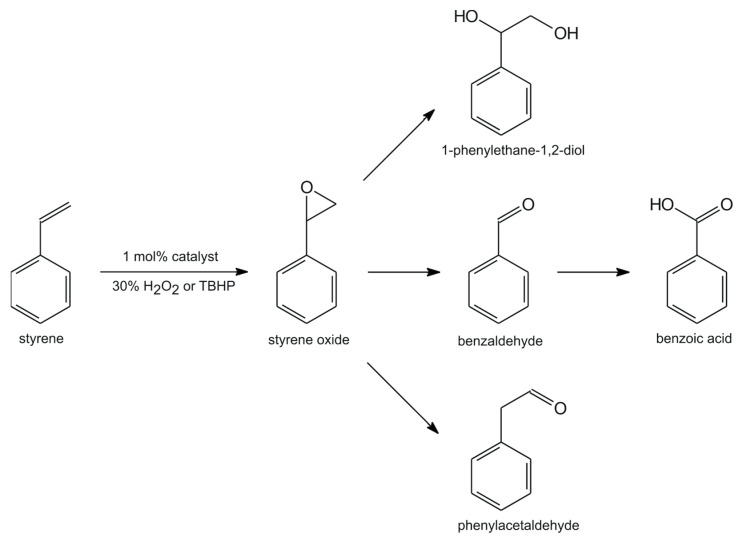
Possible products of catalytic oxidation of styrene.

The oxidation of styrene using 30% H_2_O_2_ as the oxidant aqueous or TBHP as the terminal oxidant generally can lead to five oxidation products, as was concluded by our previous observations [42], i.e., styrene oxide, phenylacetaldehyde, 1-phenylethane-1,2-diol, benzaldehyde, and benzoic acid (Figure 3). In the first step of the epoxidation reaction, styrene oxide is usually formed. Furthermore, its very fast conversion, via a nucleophilic attack of an excess of oxidant and the cleavage of hydroperoxystyrene as an intermediate product, results in the formation of benzaldehyde [43]. On the other hand, a radical mechanism can be responsible for the oxidative cleavage of the styrene side-chain double bond and the direct formation of benzaldehyde and its further oxidation to benzoic acid. The presence of water in the oxidant, such as 30% H_2_O_2_, can be blamed for the very low conversion of styrene but also for the hydrolysis of styrene oxide, resulting in the formation of 1-phenylethane-1,2-diol and the decomposition of the catalyst. Moreover, the formation of phenylacetaldehyde is also possible by way of styrene oxide isomerization.

In Table 1, the results of the oxidation of styrene by aqueous 30% H_2_O_2_ as the terminal oxidant with catalytic amounts of **[(+)VOL^1^]** and **[(−)VOL^1^]** are presented, which have shown only low conversions (25 and 27%) but with higher selectivity towards styrene oxide than benzaldehyde. The selectivity towards styrene oxide significantly decreases with TBHP, and the conversion of styrene increased to 86–96%. The formation of styrene oxide and benzaldehyde as major products was observed with both oxidants but without considerable amounts of any by-products. Our previous studies with other vanadium(V) catalysts with chiral amino alcohol-derived tridentate Schiff bases have led to similar outcomes [40,42].

Recent reports in the catalytic epoxidation of styrene by vanadium(V) complexes with Schiff base ligands, i.e., [VO_2_(sal-aebmz)], [VO_2_(sal-ambmz)], [VO_2_(acac-ambmz)] [44], and [VO(hap-dahp)] [45], have shown low selectivity (2–11%) and conversion from 51 to 78% for the most desired product, i.e., styrene oxide, and the formation of benzaldehyde in high amounts with 30% H_2_O_2_ as the terminal oxidant, even 90% in the case of [VO_2_(acac-aebmz)]. In contrast to the **[(+)VOL^1-5^]** and **[(−)VOL^1-5^]** catalysts, conversion considerably decreased to as low as 20% with TBHP but selectivity was comparable. In the case of [VO_2_(acac-ambmz)], the change was even up to 47% towards styrene oxide. Moreover, a comparison of dioxidovanadium(V) salicylaldehyde semicarbazone complexes with different substituents in salicyl moiety shows much lower conversions using 30% H_2_O_2_ and 70% TBHP [46]. Surprisingly, when *para*-substituted styrene derivatives were used as substrates, no product formation was observed. The best results for catalytic oxidation of alkenes were found for oxidovanadium(V) complexes with tridentate Schiff bases [38].

**Figure 4:**
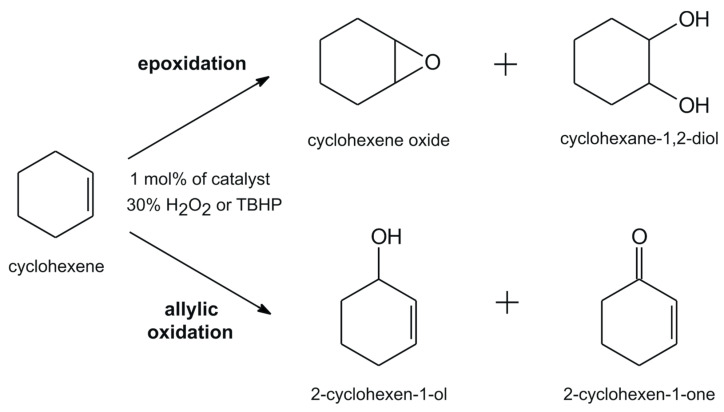
Allylic oxidation and epoxidation products of cyclohexene.

The catalytic potential of **[(+)VOL^1-5^]** and **[(−)VOL^1-5^]** complexes has also been studied for the oxidation of cyclohexene by aqueous 30% H_2_O_2_ or *tert*-butyl hydroperoxide (TBHP), resulting in the epoxidation products, i.e., cyclohexene oxide and cyclohexene-1,2-diol, after a continued hydrolysis reaction, as well as the formation of products of allylic oxidation, i.e., 2-cyclohexen-1-ol and 2-cyclohexen-1-one (Figure 4). In Table 2, we presented the results of the conversion of cyclohexene and the selectivity towards various oxidation products. When 30% H_2_O_2_ as the terminal oxidant was used, the **[(+)VOL^1^]** and **[(−)VOL^1^]** catalysts gave only 42 and 44% conversion, respectively, under the previously mentioned optimized reaction conditions. It is noteworthy that the cyclohexene oxidation with H_2_O_2_, reported for the oxidovanadium(V) Schiff base complex and derived from 3-hydroxy-2 naphthohydrazide [47], have shown distinctly higher conversion (92%) and selectivity towards the allylic oxidation product, cyclohexen-1-ol (55%), but low amounts of cyclohexene oxide (29%). In a comparison with the series of vanadium(V) complexes, i.e., [VO(μ_2_-OCH_3_)(L^1^)]_2_, [VO_2_(L^2^)], H_2_O, and [VO_2_(L^3^)] with hydrazone ONO and NNS donor Schiff base ligands, a similar outcome was achieved [48]. In contrast to H_2_O_2_, conversions were considerably higher, up to 92%, when TBHP was used with catalytic amounts of **[(+)VOL^1-5^]** and **[(−)VOL^1-5^]**, and the selectivity was higher towards 2-cyclohexene-1-one formation than cyclohexene oxide. The preferential attack of the activated C‒H bond over the C=C bond may be responsible for the creation of allylic oxidation products in higher selectivity [49].

Naturally occurring mono- or bicyclic monoterpenes with a cyclohexene ring, i.e., *S*(−)-limonene and (−)-α-pinene, used in these catalytic studies gave analogous epoxidation and allylic oxidation products. In comparison to cyclohexene, slightly lower conversions were obtained for *S*(−)-limonene oxidation by 30% H_2_O_2_; the main reaction product was epoxide, up to 75%. Surprisingly, the formation of allylic oxidation products was not observed, but significant amounts of diepoxide as a by-product may be created due to additional exocyclic isopropenyl fragments, which are present in this monoterpene (Table 3). When TBHP was used as the oxygen source, lower amounts of *S*(−)-limonene oxide were observed, but with excellent conversions, even up to 100%. The oxidation of (−)-α-pinene has given distinctly lower conversions than in the case of *S*(−)-limonene (Table 4), but significantly higher selectivity was found towards (−)-α-pinene oxide (up to 83% with TBHP and 86% with 30% H_2_O_2_ in the case of **[(−)VOL^1^]**).

### 2.5. Biological Studies

Oxidovanadium(V) complexes **[(+)VOL^1^]**, **[(+)VOL^3^]**, **[(+)VOL^4^]**, **[(−)VOL^3^],** and **[(−)VOL^4^]** have been studied to establish their cytotoxic and cytoprotective abilities on the hippocampal neuronal cell line HT-22. Moreover, for comparison, the *cis*-dioxidomolybdenum(VI) Schiff base complexes derived from the same chiral tetradentate amino alcohols [50], i.e., **[(+)MoO_2_(HL^1^)(CH_3_OH)]** and **[(+)MoO_2_(HL^3^)]**, were also used in this study.

The concentration-dependent effect of the oxidovanadium(V) and dioxidomolybdenum(VI) compounds, as well as their cytotoxicity, on the viability of the hippocampal neuronal cell line HT-22 in the range of 10–100 μM concentration, has been investigated, as well as their cytoprotective ability against 500 µM H_2_O_2_-induced oxidative damage [51].

**Figure 5:**
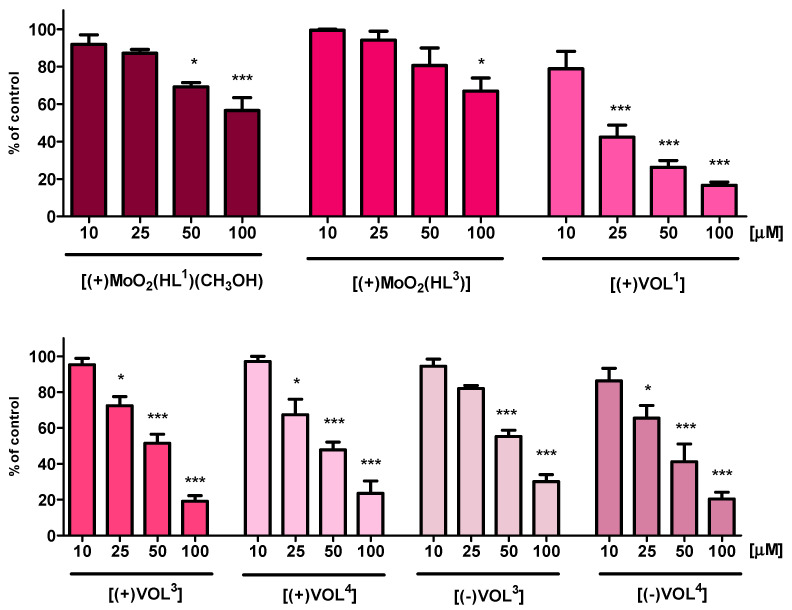
MTT assay after 24 h exposure to test compounds showing the viability of HT-22 cell line. Data are expressed as mean ± SD values from three experiments. * *p* < 0.05, *** *p* < 0.001, as compared to control (untreated) cells.

The MTT assay results of oxidovanadium(V) and *cis*-dioxidomolybdenum(VI) tetradentate Schiff base complexes were compared and showed different influences in their cytotoxicity on the HT-22 cell line (Figure 5). In the case of **[(+)MoO_2_(HL^1^)(CH_3_OH)]** and **[(+)MoO_2_(HL^3^)]**, their 10 and 25 μM concentrations have not exhibited cytotoxic effects on the viability of tested cells. Furthermore, it has been observed that a 50 μM concentration reduces the viability of cells below 70 and 80%, respectively, and their 100 μM concentrations lead to a larger decrement, even to 55% in the case of **[(+)MoO_2_(HL^1^)(CH_3_OH)]**. The MTT assay for **[(+)VOL^3^]**, **[(+)VOL^4^]**, **[(−)VOL^3^]**, and **[(−)VOL^4^]** have disclosed no significant impact on the viability of HT-22 cell line only at a 10 μM dose, although in case of **[(+)VOL^1^]**, there was decrease below 80%. Higher concentrations of vanadium(V) compounds caused a further decrease in viability, even below 30% for 100 μM doses, which revealed their higher cytotoxic effect in comparison to *cis*-dioxidomolybdenum(VI) Schiff base complexes.

**Figure 6:**
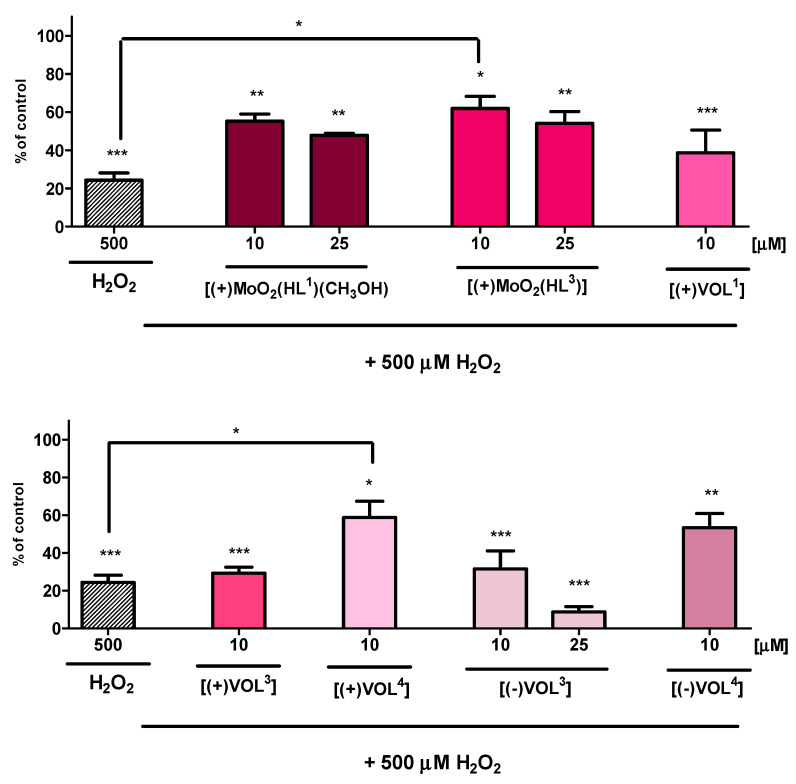
MTT assay after 24 h exposure to test compounds and 500 µM H_2_O_2_ showing the viability of HT-22 cell line. Data are expressed as mean ±SD values from three experiments. * *p* < 0.05, ** *p* < 0.01, *** *p* < 0.001, as compared to control (untreated) cells and to 500 µM H_2_O_2_.

Cytoprotective activity experiments of all complexes were performed with a 500 µM dose of 30% H_2_O_2_ to induce a cellular injury in the hippocampal cells. After 24 h, exposure of HT-22 cells to such an amount of H_2_O_2_ results in cellular death of about 24% due to oxidative stress (Figure 6). **[(+)MoO_2_(HL^1^)(CH_3_OH)]** and **[(+)MoO_2_(HL^3^)]**, which exhibited no significant cytotoxicity at 10 and 25 μM concentrations, have been tested only in these doses in experiments revealing their cytoprotective properties. These *cis*-dioxidomolybdenum(VI) complexes have shown strong cytoprotective properties, causing the highest increase, up to 62%, in the viability of HT-22 cells (10 μM dose) and 55% (25 μM dose) for **[(+)MoO_2_(HL^3^)]**. Furthermore, the addition of **[(+)MoO_2_(HL^1^)(CH_3_OH)]** with 500 µM H_2_O_2_ has a weaker influence on the viability of hippocampal cells, i.e., 57% at a 10 μM dose and 49% at a 25 μM dose.

In the screening of cytotoxic effects of **[(+)VOL^1^]**, **[(+)VOL^3^]**, **[(+)VOL^4^]**, **[(−)VOL^3^]**, and **[(−)VOL^4^]**, no considerable effect on the viability of HT-22 cells at only a 10 μM concentration was observed. It is noteworthy that only **[(−)VOL^3^]** did not distincly decrease the viability at a 25 μM dose, just slightly below 80%. On the contrary, potassium bisperoxo(1,10-phenanthroline)oxovanadate complex (BpV), which is a specific inhibitor of protein tyrosine phosphatases, has shown much higher cytotoxicity after 24 h with below 60 and even 20% cell viability at 3 and 30 μM concentrations, respectively [52]. All investigated oxidovanadium(V) complexes exhibited a cytoprotective effect against oxidative damage only at 10 μM doses. Unexpectedly, in contrast to *cis*-dioxidomolybdenum(VI) complexes, the weakest cytoprotective properties have been found in the case of **[(+)VOL^3^]** and **[(−)VOL^3^]** without any significant differences between these oxidovanadium(V) complexes with Schiff base possessing opposite chirality. Moreover, **[(−)VOL^3^]** at a 25 μM concentration did not show any cytoprotective activity. The highest cytoprotection at the mitochondrial level in the MTT test occurred for **[(+)VOL^4^]**, with up to 60% of HT-22 cell viability for a 10 μM dose. The higher cytotoxicity of vanadium(V) complexes is very likely caused by the formation of peroxidovanadium(V) species after the addition of 30% H_2_O_2_, in comparison to *cis*-dioxidomolybdenum(VI) Schiff base complexes derived from the same chiral tetradentate amino alcohols and can be the reason for the stronger harmful effect on hippocampal cells [53]. Such properties of various vanadium compounds may emphasize their importance as promising drug candidates in chemotherapy and targeted cancer therapies, with possible relatively strong cytotoxicity and antimetastatic activity [54].

**Figure 7:**
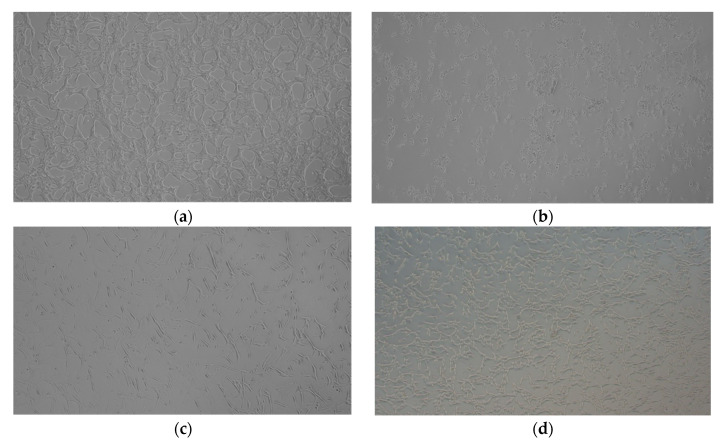
HT-22 cell morphology: (**a**) control cells and after exposure to (**b**) 500 µM of 30% H_2_O_2_, (**c**) 10 µM **[(+)MoO_2_(HL^3^)]** and 500 µM of 30% H_2_O_2_, and (**d**) 10 µM of **[(+)VOL^4^]** and 500 µM of 30% H_2_O_2_.

Morphological changes and cell survival in HT-22 cells have been observed on microscopic images comparing control cells (Figure 7a) with the same cells after treatment at 30% H_2_O_2_ (500 µM), which manifest themselves in irregular shapes of the hippocampal cells and shrink (Figure 7b). This is also true for the same cells pre-treated with *cis*-dioxidomolybdenum(VI) (Figure 7c) and oxidovanadium(V) (Figure 7d) complexes, which show the best cytoprotective ability.

## 3. Materials and Methods

### 3.1. Measurements

All reagents for synthesis of the complexes, i.e., chiral amino alcohols, salicylaldehyde derivatives, and vanadium(V) oxytripropoxide were purchased from Aldrich company. The other chemicals were obtained from local sources and used without further purification. Carlo Erba MOD 1106 instrument was used to perform elemental analyses. UV-Vis spectra were conducted using a Perkin-Elmer LAMBDA 18 spectrophotometer, and CD spectra were conducted using a Jasco J-815 spectropolarimeter. IR spectra were recorded on Bruker IFS 66 as KBr pellets. Bruker AVANCE III 700 MHz spectrometer was employed for all NMR spectra measurements using TMS as a reference and DMSO-*d_6_* as a solvent. The catalytic reactions progress was monitored on a Shimadzu GC-2025 gas chromatograph with an FID detector, and Zebron ZB-5 capillary column and Shimadzu GCMS-QP2010 SE equipment were used for the confirmation of the identities of all oxidation products. The absorbance of the cells during the MTT assay was measured at 570 nm and 660 nm (reference value) by an ASYS Hitech GmbH microplate reader. Microscopic images were taken at 1 × 4 magnification with an Olympus microscope.

### 3.2. Synthesis of Oxidovanadium(V) Complexes

All vanadium(V) complexes were synthesized employing the same procedure. To a solution of 1 mmol of chiral amino alcohol, i.e., *R*(+)-3-amino-1,2-propanediol or *S*(−)-3-amino-1,2-propanediol in MeOH (10 mL), 1 mmol of one of the following aromatic *o*-hydroxyaldehyde, i.e., 3-methoxysalicylaldehyde, 5-methoxysalicylaldehyde, 5-methylsalicylaldehyde, 5-bromosalicylaldehyde, or 5-nitrosalicylaldehyde in 10 mL of MeOH, was added. Then, reaction mixture was stirred and heated under reflux for 1 h. After addition of vanadium(V) oxytripropoxide (1 mmol) in MeOH (10 mL), reactants in solution were stirred under reflux for next 2 h. Obtained precipitates were filtered off and washed several times with cold MeOH. All complexes are crystalline solids (Figure S5).

***[(+)VOL^1^]***: Yield 83%, brown. *Anal.* Calc. for C_11_H_12_NO_5_V: C, 45.7; H, 4.2; N, 4.8. Found: C, 45.8; H, 4.1; N, 4.8%. IR (KBr, cm^−1^): 1642 (ν_C=N_); 1598 (ν_C=C_); 1286, 1082 (ν_C-O_); 968 (ν_V=O_). UV-Vis spectrum in DMSO [λ_max_ (nm), ɛ (M^−1^ cm^−1^)]: 351 (3200). CD spectrum in DMSO [λ_max_ (nm), Δɛ (M^−1^ cm^−1^)]: 280 (−0.40), 305 (4.19), 374 (1.97). ^1^H NMR (DMSO-*d_6_*, ppm): 8.69 (1H, s) (azomethine); 7.16 (1H, d, ^3^*J* = 8.4 Hz), 7.13 (1H, d, ^3^*J* = 7.8 Hz), 6.87 (1H, t, ^3^*J* = 8.4 Hz) (aromatic); 5.16 (1H, m) (methine); 5.42 (1H, dd, ^3^*J* = 11.2 Hz, ^4^*J* = 2.8 Hz), 4.86 (1H, dd, ^3^*J* = 11.2 Hz, ^4^*J* = 5.5 Hz), 4.72 (1H, dd, ^3^*J* = 13.1 Hz, ^4^*J* = 5.5 Hz), 3.73 (1H, dd, ^3^*J* = 13.1 Hz, ^4^*J* = 5.5 Hz) (methylene), 3.82 (3H, s) (methoxy). ^51^V NMR (DMSO-*d*_6_, ppm): −531.8.

***[(+)VOL^2^]***: Yield 78%, brown. *Anal.* Calc. for C_11_H_12_NO_5_V: C, 45.7; H, 4.2; N, 4.8. Found: C, 45.7; H, 4.3; N, 4.7%. IR (KBr, cm^−1^): 1650 (ν_C=N_); 1612 (ν_C=C_); 1293, 1095 (ν_C-O_); 980 (ν_V=O_). UV-Vis spectrum in DMSO [λ_max_ (nm), ɛ (M^−1^ cm^−1^)]: 359 (3540). CD spectrum in DMSO [λ_max_ (nm), Δɛ (M^−1^ cm^−1^)]: 280 (−0.40), 310 (3.86), 379 (1.53). ^1^H NMR (DMSO-*d_6_*, ppm): 8.68 (1H, s) (azomethine); 7.18 (1H, d, ^4^*J* = 2.8 Hz), 7.15 (1H, dd, ^3^*J* = 8.8 Hz, ^4^*J* = 2.8 Hz), 6.84 (1H, d, ^3^*J* = 8.8 Hz) (aromatic); 5.18 (1H, m) (methine); 5.40 (1H, dd, ^3^*J* = 11.2 Hz, ^4^*J* = 2.8 Hz), 4.84 (1H, dd, ^3^*J* = 11.2 Hz, ^4^*J* = 5.5 Hz), 4.71 (1H, dd, ^3^*J* = 13.1 Hz, ^4^*J* = 5.5 Hz), 3.72 (1H, dd, ^3^*J* = 13.1 Hz, ^4^*J* = 5.5 Hz) (methylene), 3.81 (3H, s) (methoxy). ^51^V NMR (DMSO-*d_6_*, ppm): −532.9.

***[(+)VOL^3^]***: Yield 85%, dark red. *Anal.* Calc. for C_11_H_12_NO_4_V: C, 48.4; H, 4.4; N, 5.1. Found: C, 48.2; H, 4.5; N, 5.1%. IR (KBr, cm^−1^): 1640 (ν_C=N_); 1608 (ν_C=C_); 1286, 1058 (ν_C-O_); 979 (ν_V=O_). UV-Vis spectrum in DMSO [λ_max_ (nm), ɛ (M^−1^ cm^−1^)]: 339 (3480). CD spectrum in DMSO [λ_max_ (nm), Δɛ (M^−1^ cm^−1^)]: 279 (−2.10), 309 (3.33), 370 (2.89). ^1^H NMR (DMSO-*d_6_*, ppm): 8.69 (1H, s) (azomethine); 7.38 (1H, s), 7.31 (1H, d, ^3^*J* = 9.2 Hz), 6.84 (1H, d, ^3^*J* = 8.3 Hz) (aromatic); 5.15 (1H, m) (methine); 5.35 (1H, dd, ^3^*J* = 11.0 Hz, ^4^*J* = 2.7 Hz), 4.86 (1H, dd, ^3^*J* = 11.0 Hz, ^4^*J* = 5.5 Hz), 4.71 (1H, dd, ^3^*J* = 13.0 Hz, ^4^*J* = 5.5 Hz), 3.81 (1H, dd, ^3^*J* = 13.0 Hz, ^4^*J* = 5.5 Hz) (methylene). 2.31 (3H, d, ^3^*J* = 5.3) (methyl). ^51^V NMR (DMSO-*d*_6_, ppm): −532.5.

***[(+)VOL^4^]***: Yield 80%, orange. *Anal.* Calc. for C_10_H_9_BrNO_4_V: C, 35.5; H, 2.7; N, 4.1. Found: C, 35.6; H, 2.7; N, 4.0%. IR (KBr, cm^−1^): 1646 (ν_C=N_); 1587 (ν_C=C_); 1280, 1048 (ν_C-O_); 980 (ν_V=O_). UV-Vis spectrum in DMSO [λ_max_ (nm), ɛ (M^−1^ cm^−1^)]: 340 (2860). CD spectrum in DMSO [λ_max_ (nm), Δɛ (M^−1^ cm^−1^)]: 281 (−1.47), 322 (1.79), 367 (2.67). ^1^H NMR (DMSO-*d_6_*, ppm): 8.70 (1H, s) (azomethine); 7.71 (1H, d, ^3^*J* = 2.6 Hz), 7.52 (1H, dd, ^3^*J* = 8.9 Hz, ^4^*J* = 2.6 Hz), 6.71 (1H, d, ^3^*J* = 8.9 Hz) (aromatic); 5.19 (1H, m) (methine); 5.44 (1H, dd, ^3^*J* = 11.2 Hz, ^4^*J* = 2.8 Hz), 4.89 (1H, dd, ^3^*J* = 11.2 Hz, ^4^*J* = 5.5 Hz), 4.74 (1H, dd, ^3^*J* = 13.1 Hz, ^4^*J* = 5.5 Hz), 3.75 (1H, dd, ^3^*J* = 13.1 Hz, ^4^*J* = 5.5 Hz) (methylene). ^51^V NMR (DMSO-*d*_6_, ppm): −531.7.

***[(+)VOL^5^]***: Yield 88%, brown. *Anal.* Calc. for C_10_H_9_N_2_O_6_V: C, 39.5; H, 3.0; N, 9.2. Found: C, 39.4; H, 3.1; N, 9.3%. IR (KBr, cm^−1^): 1641 (ν_C=N_); 1607 (ν_C=C_); 1558, 1339 (ν_NO2_); 1316, 1049 (ν_C-O_); 976 (ν_V=O_). UV-Vis spectrum in DMSO [λ_max_ (nm), ɛ (M^−1^ cm^−1^)]: 355 (12060). CD spectrum in DMSO [λ_max_ (nm), Δɛ (M^−1^ cm^−1^)]: 279 (-2.03), 304 (3.16), 390 (2.74). ^1^H NMR (DMSO-*d_6_*, ppm): 8.68 (1H, s) (azomethine); 8.46 (1H, dd, ^3^*J* = 8.4 Hz, ^4^*J* = 2.9 Hz), 8.28 (1H, dd, ^3^*J* = 8.4 Hz, ^4^*J* = 2.9 Hz), 6.96 (1H, d, ^3^*J* = 8.4 Hz) (aromatic); 5.36 (1H, m) (methine); 5.48 (1H, dd, ^3^*J* = 11.2 Hz, ^4^*J* = 2.8 Hz), 4.92 (1H, dd, ^3^*J* = 11.2 Hz, ^4^*J* = 5.5 Hz), 4.75 (1H, dd, ^3^*J* = 13.1 Hz, ^4^*J* = 5.5 Hz), 3.77 (1H, dd, ^3^*J* = 13.1 Hz, ^4^*J* = 5.5 Hz) (methylene). ^51^V NMR (DMSO-*d_6_*, ppm): −529.8.

***[(−)VOL^1^]***: Yield 87%, brown. *Anal.* Calc. for C_11_H_12_NO_5_V: C, 45.7; H, 4.2; N, 4.8. Found: C, 45.6; H, 4.2; N, 4.8%. IR (KBr, cm^−1^): 1643 (ν_C=N_); 1599 (ν_C=C_); 1277, 1081 (ν_C-O_); 956 (ν_V=O_). UV-Vis spectrum in DMSO [λ_max_ (nm), ɛ (M^−1^ cm^−1^)]: 350 (3360). CD spectrum in DMSO [λ_max_ (nm), Δɛ (M^−1^ cm^−1^)]: 278 (1.53), 307 (−6.70), 362 (−2.27).

***[(−)VOL^2^]***: Yield 82%, brown. *Anal.* Calc. for C_11_H_12_NO_5_V: C, 45.7; H, 4.2; N, 4.8. Found: C, 45.6; H, 4.3; N, 4.8%. IR (KBr, cm^−1^): 1650 (ν_C=N_); 1612 (ν_C=C_); 1278, 1084 (ν_C-O_); 998 (ν_V=O_). UV-Vis spectrum in DMSO [λ_max_ (nm), ɛ (M^−1^ cm^−1^)]: 358 (3420). CD spectrum in DMSO [λ_max_ (nm), Δɛ (M^−1^ cm^−1^)]: 280 (0.98), 310 (−3.72), 379 (−1.43).

***[(−)VOL^3^]***: Yield 86%, dark red. *Anal.* Calc. for C_11_H_12_NO_4_V: C, 48.4; H, 4.4; N, 5.1. Found: C, 48.3; H, 4.4; N, 5.0%. IR (KBr, cm^−1^): 1641 (ν_C=N_); 1584 (ν_C=C_); 1286, 1058 (ν_C-O_); 978 (ν_V=O_). UV-Vis spectrum in DMSO [λ_max_ (nm), ɛ (M^−1^ cm^−1^)]: 340 (3560). CD spectrum in DMSO [λ_max_ (nm), Δɛ (M^−1^ cm^−1^)]: 278 (2.24), 308 (−4.14), 365 (−2.58).

***[(−)VOL^4^]***: Yield 84%, orange. *Anal.* Calc. for C_10_H_9_BrNO_4_V: C, 35.5; H, 2.7; N, 4.1. Found: C, 35.4; H, 2.8; N, 4.0%. IR (KBr, cm^−1^): 1647 (ν_C=N_); 1589 (ν_C=C_); 1280, 1047 (ν_C-O_); 981 (ν_V=O_). UV-Vis spectrum in DMSO [λ_max_ (nm), ɛ (M^−1^ cm^−1^)]: 342 (2940). CD spectrum in DMSO [λ_max_ (nm), Δɛ (M^−1^ cm^−1^)]: 280 (1.34), 318 (−2.18), 366 (−2.48).

***[(−)VOL^5^]***: Yield 81%, brown. *Anal.* Calc. for C_10_H_9_N_2_O_6_V: C, 39.5; H, 3.0; N, 9.2. Found: C, 39.6; H, 3.1; N, 9.2%. IR (KBr, cm^−1^): 1652 (ν_C=N_); 1607 (ν_C=C_); 1560, 1336 (ν_NO2_); 1305, 1046 (ν_C-O_); 956 (ν_V=O_). UV-Vis spectrum in DMSO [λ_max_ (nm), ɛ (M^−1^ cm^−1^)]: 355 (12330). CD spectrum in DMSO [λ_max_ (nm), Δɛ (M^−1^ cm^−1^)]: 278 (1.76), 304 (−2.58), 390 (−2.13).

### 3.3. Catalytic Activity

All oxidovanadium(V) complexes have been used as catalysts in the oxidation of cyclohexene, styrene, *S*(−)-limonene, and (−)-α-pinene using as an oxidant aqueous 30% H_2_O_2_ or 5.5 M solution of *tert*-butyl hydroperoxide (TBHP) in decane. All reactions were optimized with different reaction condition parameters. The optimal reaction temperature was 80 °C, and molar ratio of catalyst, substrate, and oxidant was 0.01:1:2. The best solvent for catalytic reactions with TBHP was 1,2-dichloroethane (DCE), and acetonitrile for reaction performed with H_2_O_2_ as the terminal oxidant.

### 3.4. Biological Activity

#### 3.4.1. The Cell Culture and Treatments

The mouse hippocampal neuronal cell line HT-22 was cultured in standard conditions according to protocol described by our team previously [34,35]. Working solutions at 10, 25, 50, and 100 µM concentrations were prepared *ex tempore* in serum-free medium DMEM. The measurements of the viability and cytoprotective action of investigated compounds on HT-22 cells after 24 h incubation were tested by mitochondrial dehydrogenase activity (MTT) assay.

#### 3.4.2. MTT Assay

HT-22 cells were passaged at 8 × 10^3^ cells in well of a 96-well plate and incubated for 24 h in standard conditions. HT-22 cells were treated later for the next 24 h with selected concentrations of the investigated compounds, which were not cytotoxic. In the cytoprotective activity experiments, vanadium(V) and *cis*-dioxidomolybdenum(VI) complexes were added to the HT-22 cells for 1 h and then treated for 24 h with 500 µM H_2_O_2_. MTT assay was performed according to previously described protocol by our team [34,35].

#### 3.4.3. Microscopy

HT-22 cells were seeded in 12-well plates. The plate was cultivated for 24 h under standard conditions. HT-22 cells were treated with investigated complexes for 1 h and then, after addition of 500 µM H_2_O_2_, incubated for 24 h.

## 4. Conclusions

Within this paper, we present the synthesis of a series of oxidovanadium(V) complexes derived from chiral tetradentate Schiff bases, **[(+)VOL^1-5^]** and **[(−)VOL^1-5^]**, products of a single condensation of salicylaldehyde derivatives with *R*(+)-3-amino-1,2-propanediol or *S*(−)-3-amino-1,2-propanediol, which have been further characterized by one- and two-dimensional NMR spectroscopy, as well as IR, UV-Vis, and CD techniques.

The catalytic potential of **[(+)VOL^1-5^]** and **[(−)VOL^1-5^]** complexes have also been studied in the epoxidation of styrene, cyclohexene, *S*(−)-limonene, and (−)-α-pinene in the presence of 30% H_2_O_2_ or *tert*-butyl hydroperoxide as the terminal oxidant. The conversion of all these olefins was distinctly lower when 30% H_2_O_2_ was used, probably due to its strong oxidizing nature and the presence of water, usually responsible for the hydrolysis of epoxides and the decomposition of catalyst. Generally, the catalytic amounts of all vanadium(V) complexes revealed high conversions and selectivities towards corresponding epoxides, especially in the case of monoterpenes in a non-aqueous environment when *tert*-butyl hydroperoxide (TBHP) as the oxidant. In some cases, the highest selectivities showed catalysts with electron-donating substituents in salicyladimine moiety.

The biological cytotoxicity of oxidovanadium(V) and *cis*-dioxidomolybdenum(VI) complexes derived from the same chiral Schiff bases, as well as cytoprotection against the oxidative damage caused by H_2_O_2_ towards the hippocampal neuronal cell line HT-22, based on an assessment of mitochondrial activity in MTT tests, has also been studied. The comparison of the cytotoxic and cytoprotective results between *cis*-dioxidomolybdenum(VI) and oxidovanadium(V) complexes has shown significantly higher cytotoxic activity for the latter compounds. The best cytoprotective activity has shown *cis*-dioxidomolybdenum(VI) complexes with the 5-methyl substituent in salicylaldimine fragment and, in the case of oxidovanadium(V) compounds, with the 5-bromo electron donating group.

## Figures and Tables

**Table 1:** Styrene oxidation with oxidovanadium(V) Schiff base complexes as catalysts.

Entry	Catalyst	Conversion (%)	Oxidant	Styrene Oxide (%)	Benzaldehyde (%)
1	**[(+)VOL^1^]**	25	H_2_O_2_	58	42
2	**[(+)VOL^1^]**	95	TBHP	46	54
3	**[(+)VOL^2^]**	91	TBHP	37	63
4	**[(+)VOL^3^]**	89	TBHP	35	65
5	**[(+)VOL^4^]**	90	TBHP	43	57
6	**[(+)VOL^5^]**	86	TBHP	31	69
7	**[(−)VOL^1^]**	27	H_2_O_2_	56	44
8	**[(−)VOL^1^]**	96	TBHP	48	52
9	**[(−)VOL^2^]**	92	TBHP	52	48
10	**[(−)VOL^3^]**	93	TBHP	38	62
11	**[(−)VOL^4^]**	95	TBHP	42	58
12	**[(−)VOL^5^]**	92	TBHP	49	51

**Table 2:** Cyclohexene oxidation with oxidovanadium(V) Schiff base complexes as catalysts.

Entry	Catalyst	Conversion (%)	Oxidant	Epoxide (%)	Alcohol (%)	Ketone (%)
1	**[(+)VOL^1^]**	42	H_2_O_2_	44	29	27
2	**[(+)VOL^1^]**	90	TBHP	22	37	42
3	**[(+)VOL^2^]**	87	TBHP	26	30	44
4	**[(+)VOL^3^]**	90	TBHP	21	37	42
5	**[(+)VOL^4^]**	87	TBHP	22	40	38
6	**[(+)VOL^5^]**	84	TBHP	33	27	40
7	**[(−)VOL^1^]**	44	H_2_O_2_	41	32	27
8	**[(−)VOL^1^]**	82	TBHP	36	21	43
9	**[(−)VOL^2^]**	79	TBHP	33	29	39
10	**[(−)VOL^3^]**	92	TBHP	30	22	48
11	**[(−)VOL^4^]**	87	TBHP	32	24	44
12	**[(−)VOL^5^]**	84	TBHP	28	26	46

**Table 3:** The oxidation of *S*(−)-limonene with oxidovanadium(V) Schiff base complexes as catalysts.

Entry	Catalyst	Conversion (%)	Oxidant	Epoxide (%)	Diepoxide (%)
1	**[(+)VOL^1^]**	34	H_2_O_2_	75	25
2	**[(+)VOL^1^]**	87	TBHP	70	30
3	**[(+)VOL^2^]**	93	TBHP	72	28
4	**[(+)VOL^3^]**	88	TBHP	70	30
5	**[(+)VOL^4^]**	92	TBHP	84	16
6	**[(+)VOL^5^]**	75	TBHP	73	27
7	**[(−)VOL^1^]**	37	H_2_O_2_	71	29
8	**[(−)VOL^1^]**	100	TBHP	57	43
9	**[(−)VOL^2^]**	99	TBHP	59	41
10	**[(−)VOL^3^]**	99	TBHP	56	44
11	**[(−)VOL^4^]**	87	TBHP	61	39
12	**[(−)VOL^5^]**	92	TBHP	75	25

**Table 4:** The oxidation of (−)-α-pinene with oxidovanadium(V) Schiff base complexes as catalysts.

**Entry**	**Catalyst**	**Conversion (%)**	**Oxidant**	**(−)-α-pinene Oxide (%)**
1	**[(+)VOL^1^]**	26	H_2_O_2_	79
2	**[(+)VOL^1^]**	77	TBHP	79
3	**[(+)VOL^2^]**	62	TBHP	77
4	**[(+)VOL^3^]**	83	TBHP	72
5	**[(+)VOL^4^]**	79	TBHP	73
6	**[(+)VOL^5^]**	76	TBHP	70
7	**[(−)VOL^1^]**	32	H_2_O_2_	86
8	**[(−)VOL^1^]**	69	TBHP	83
9	**[(−)VOL^2^]**	62	TBHP	79
10	**[(−)VOL^3^]**	87	TBHP	76
11	**[(−)VOL^4^]**	70	TBHP	73
12	**[(−)VOL^5^]**	62	TBHP	68

## Data Availability

Data are contained within the article.

## Supplementary Materials

The following supporting information can be downloaded at: https://www.mdpi.com/article/10.3390/ijms25095010/s1.
