# Supplementary material for: Oxidovanadium(V) Schiff Base Complexes Derived from Chiral 3-amino-1,2-propanediol Enantiomers: Synthesis, Spectroscopic Studies, Catalytic and Biological Activity
Source: Int J Mol Sci. 2024 May 3;25(9):5010. doi: 10.3390/ijms25095010 (PMC11084397; doi:10.3390/ijms25095010)

Figure S1. The IR spectra of oxidovanadium(V) complexes.

[(+)VOL[1]]

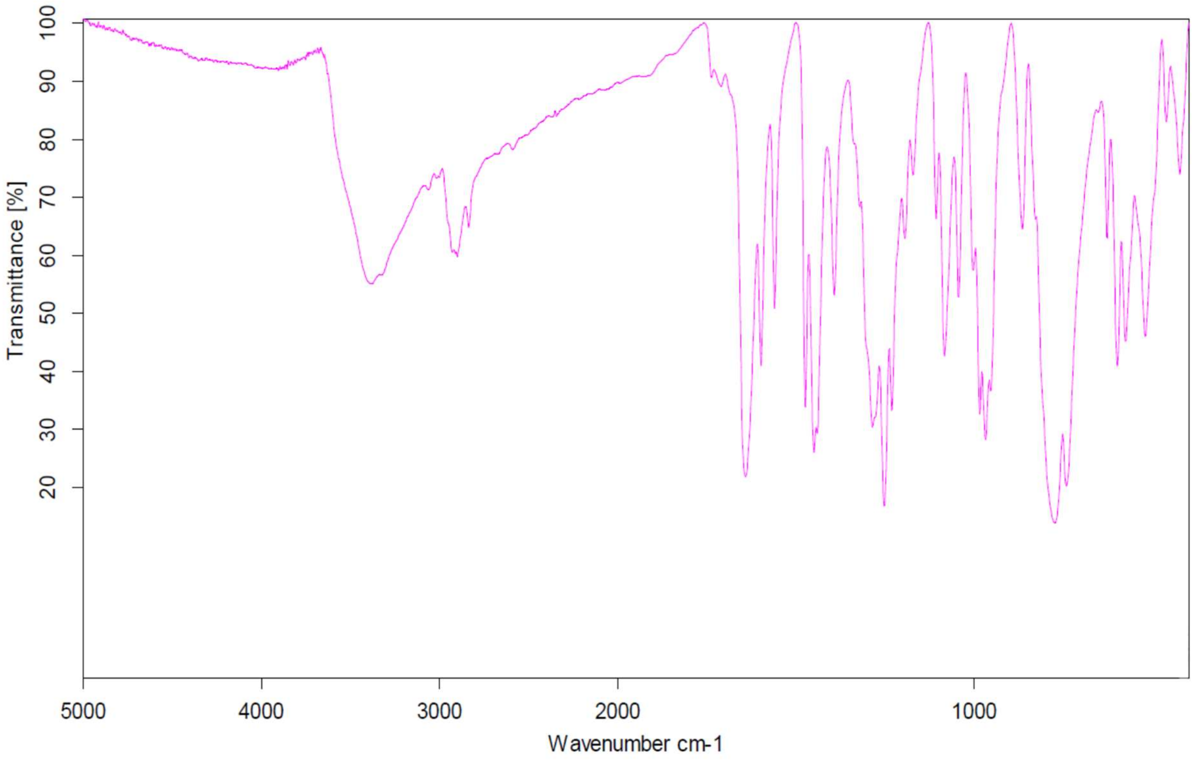

[(+)VOL[2]]

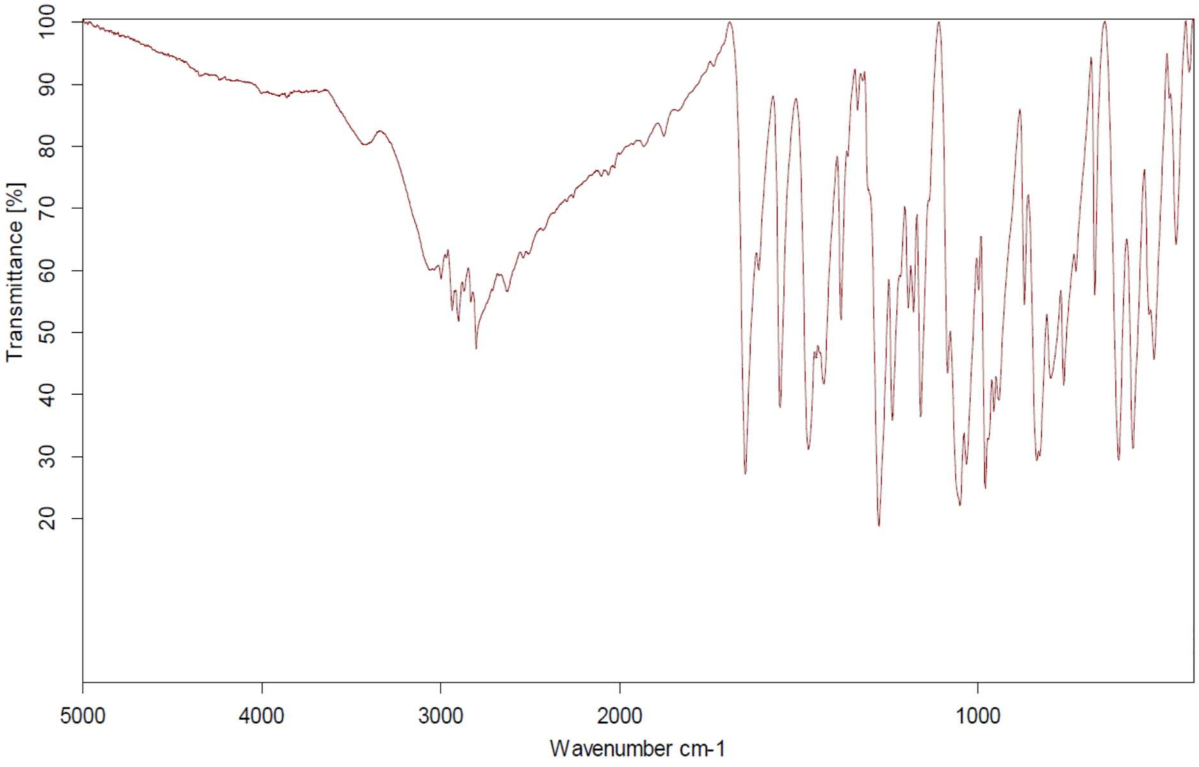

[(+)VOL[3]]

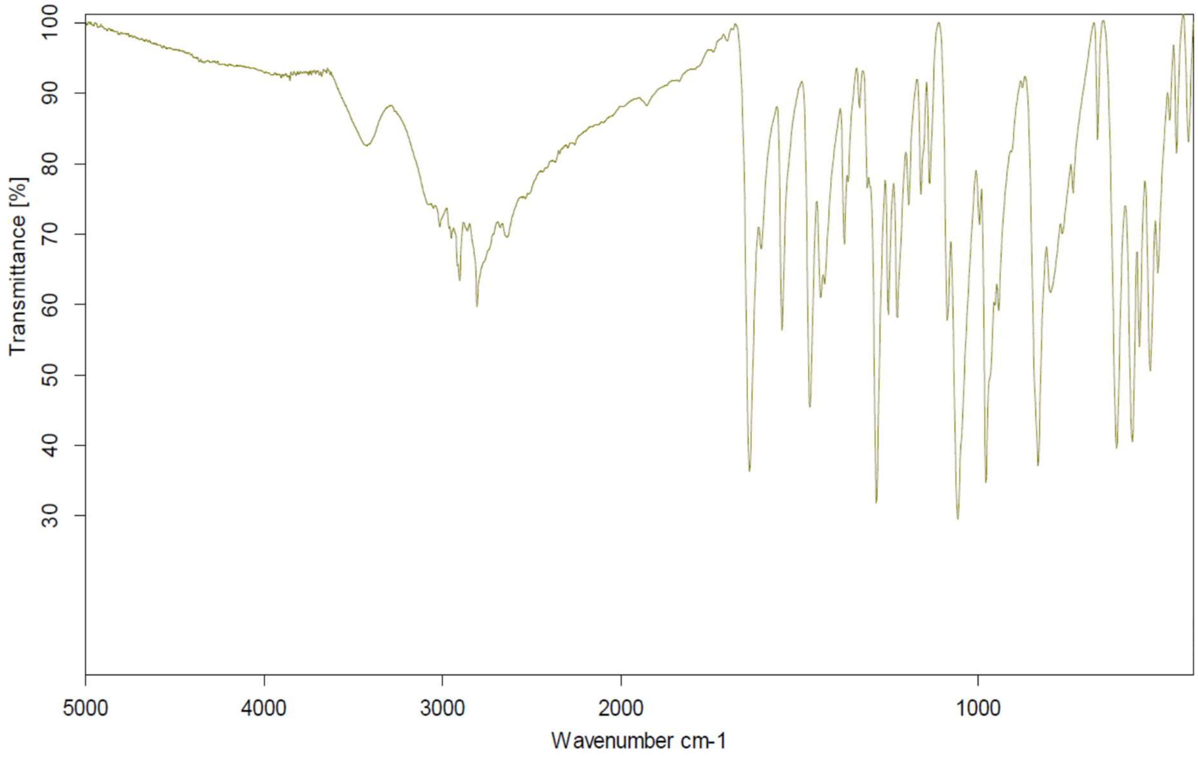
Transmittance [%]
Wavenumber cm-1
100
90
80
70
60
50
40
30
5000
4000
3000
2000
1000

[(+)VOL[4]]

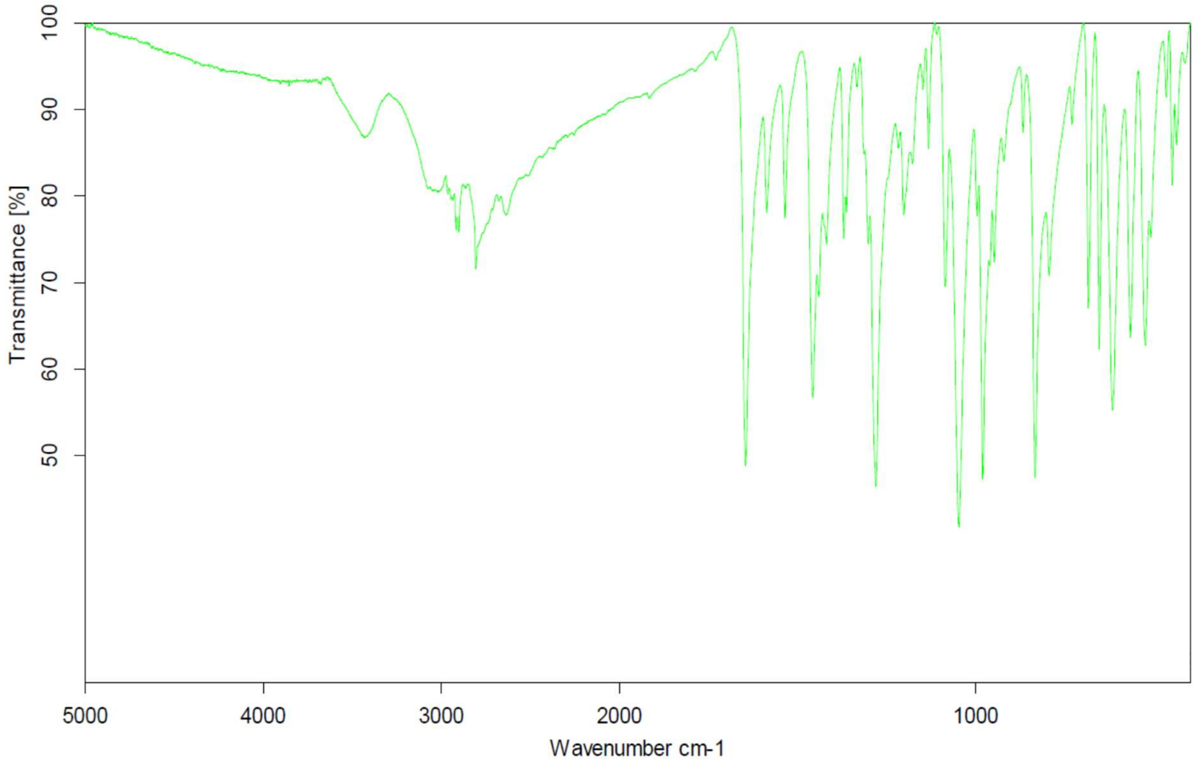
Transmittance [%]
Wavenumber cm-1
100
90
80
70
60
50
5000
4000
3000
2000
1000

[(+)VOL[5]]

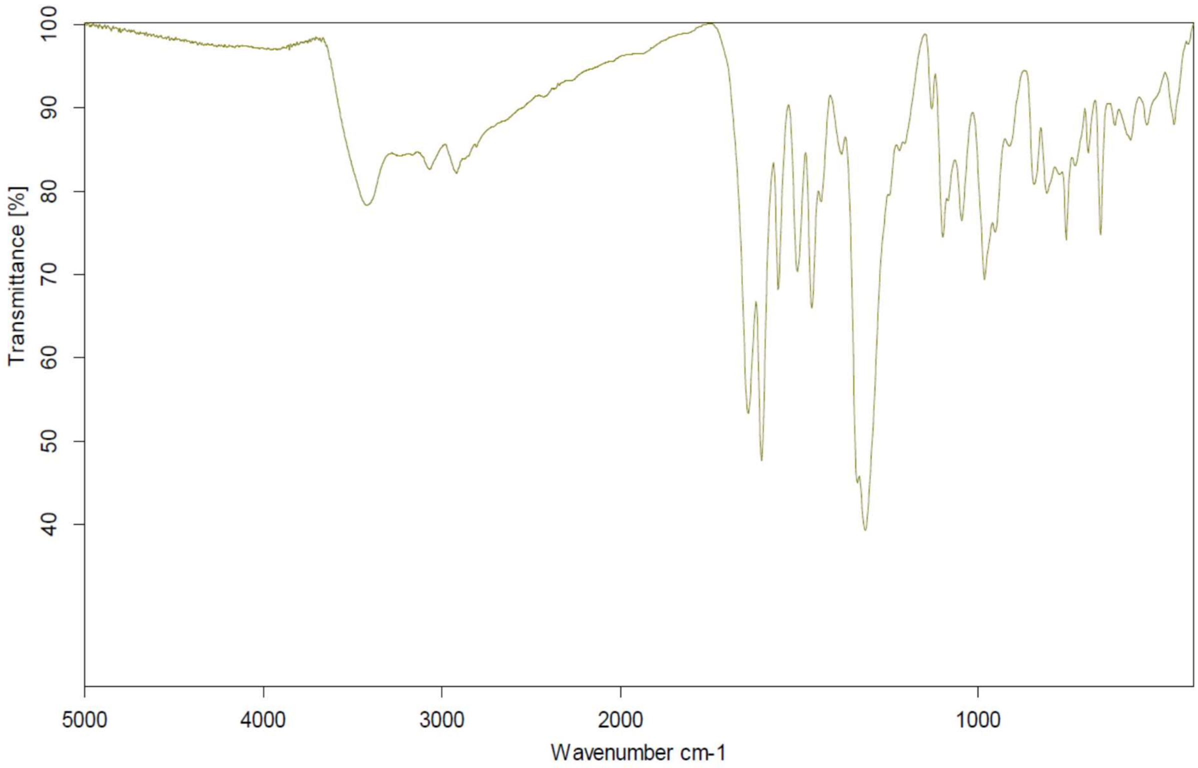
Transmittance [%]
100
90
80
70
60
50
40
5000
4000
3000
2000
1000
Wavenumber cm-1

[(-)VOL[1]]

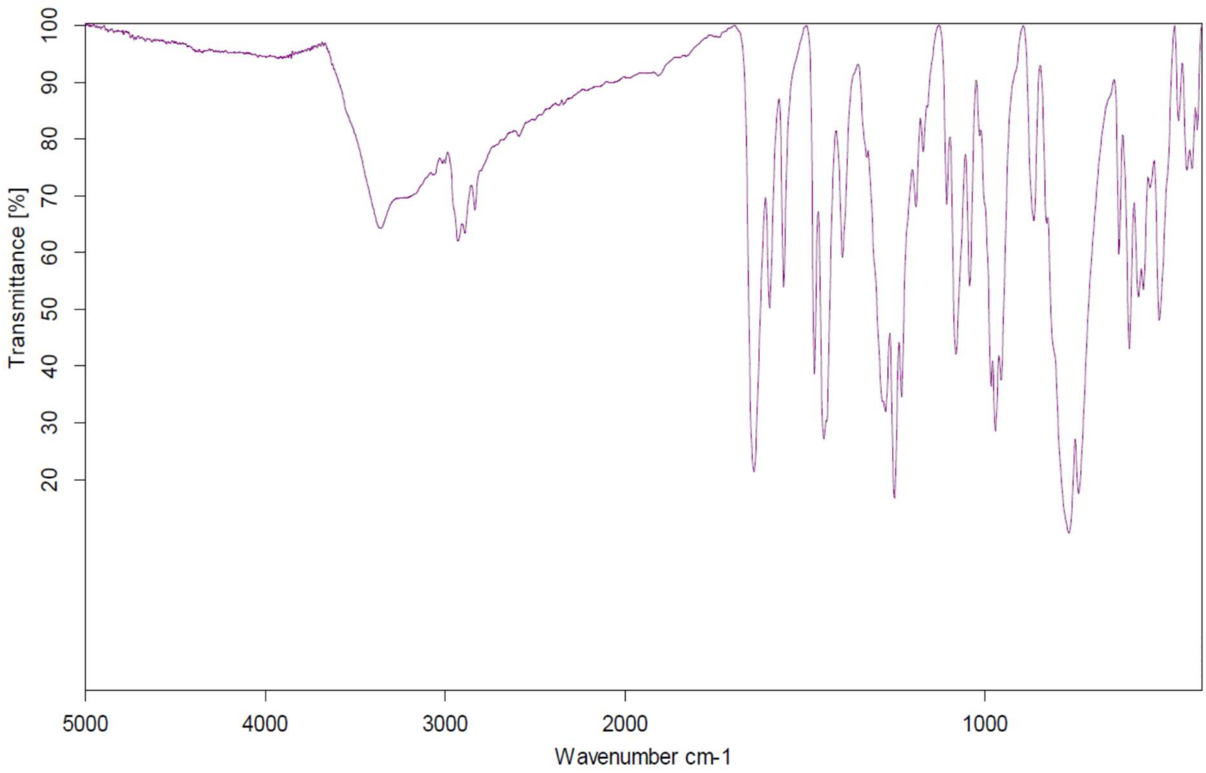
Transmittance [%]
100
90
80
70
60
50
40
30
20
5000
4000
3000
2000
1000
Wavenumber cm-1

[(-)VOL^2]

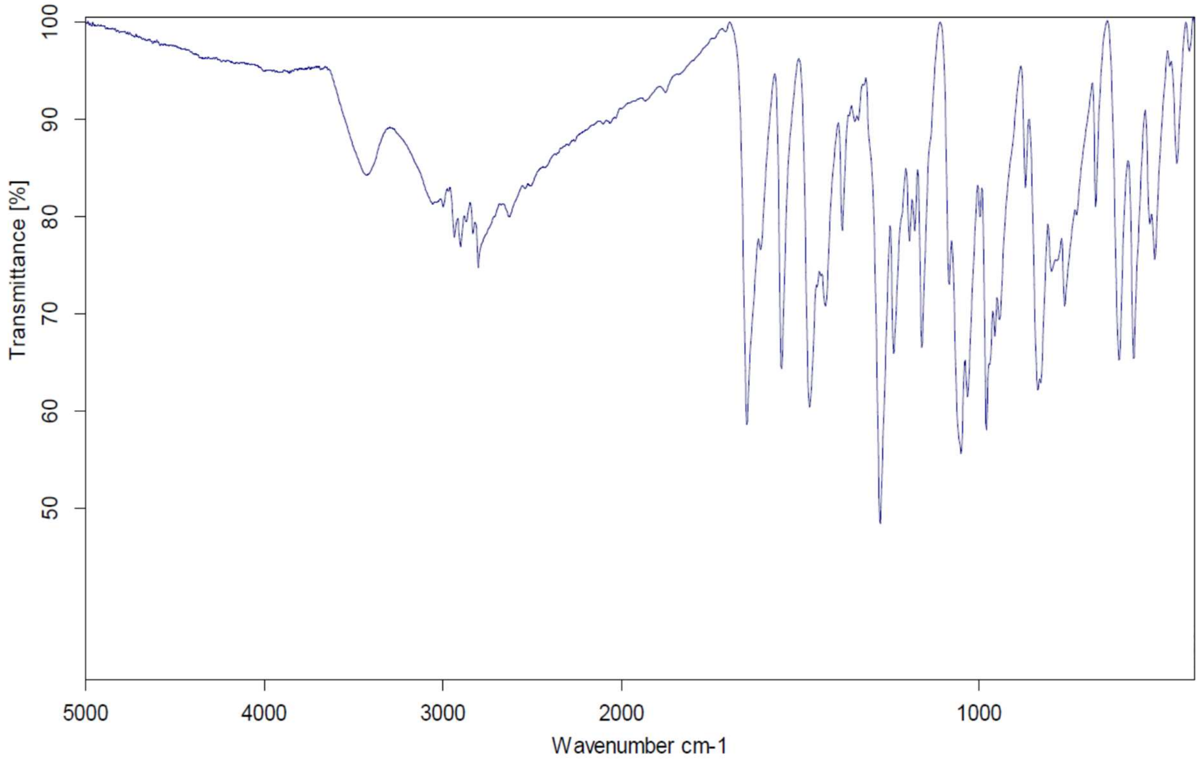
100
90
80
70
60
50
Transmittance [%]
5000
4000
3000
2000
1000
Wavenumber cm-1

[(-)VOL^3]

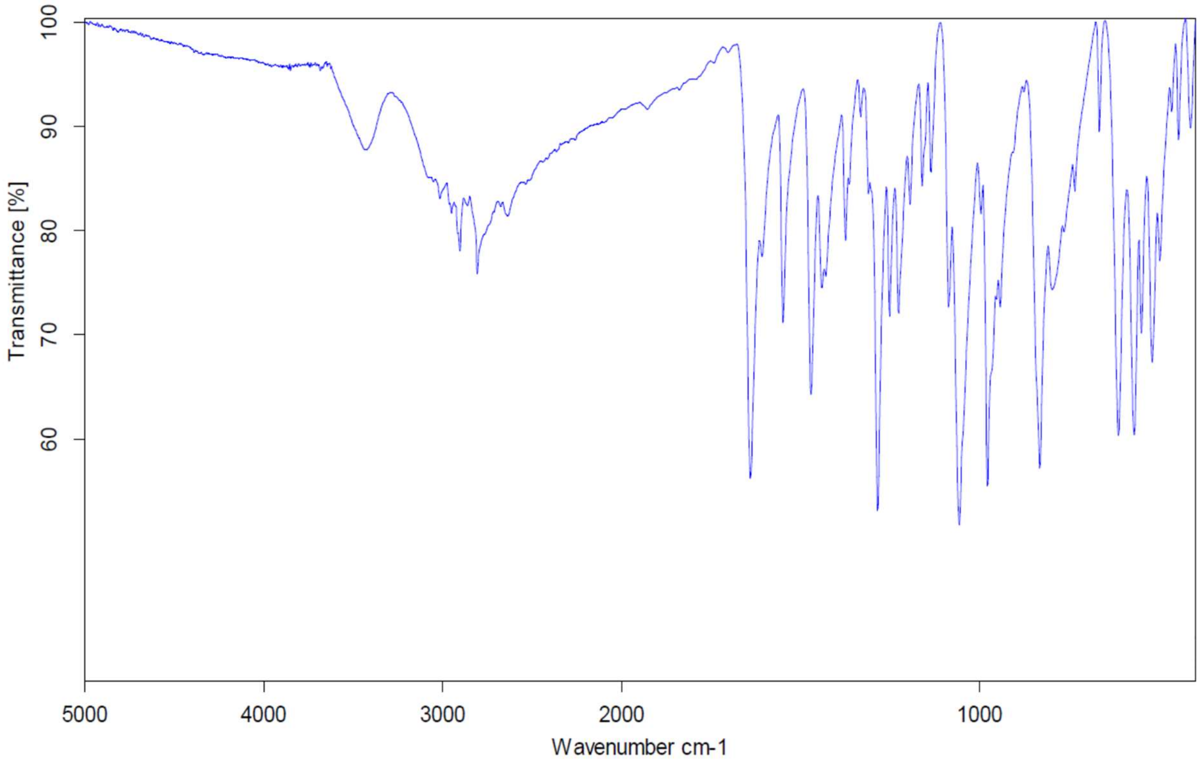
100
90
80
70
60
Transmittance [%]
5000
4000
3000
2000
1000
Wavenumber cm-1

[(-)VOL[4]]

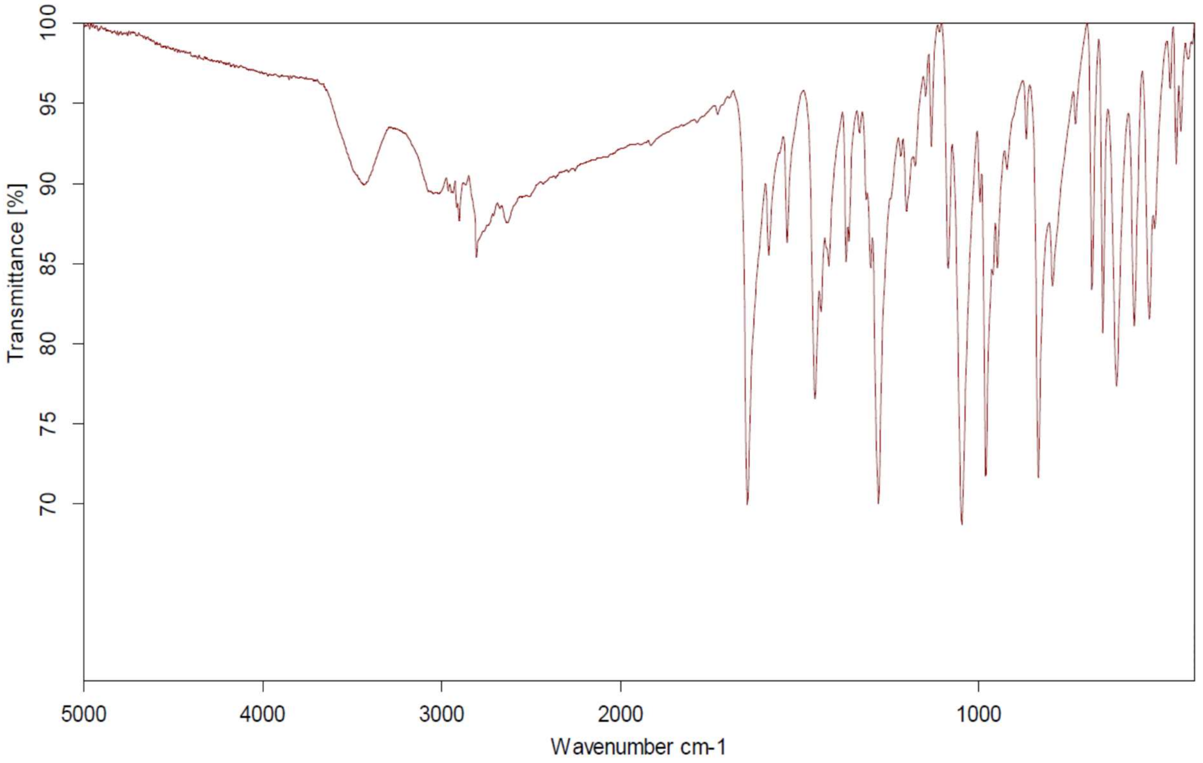

Transmittance [%]
100
95
90
85
80
75
70
5000
4000
3000
2000
1000
Wavenumber cm-1

[(-)VOL[5]]

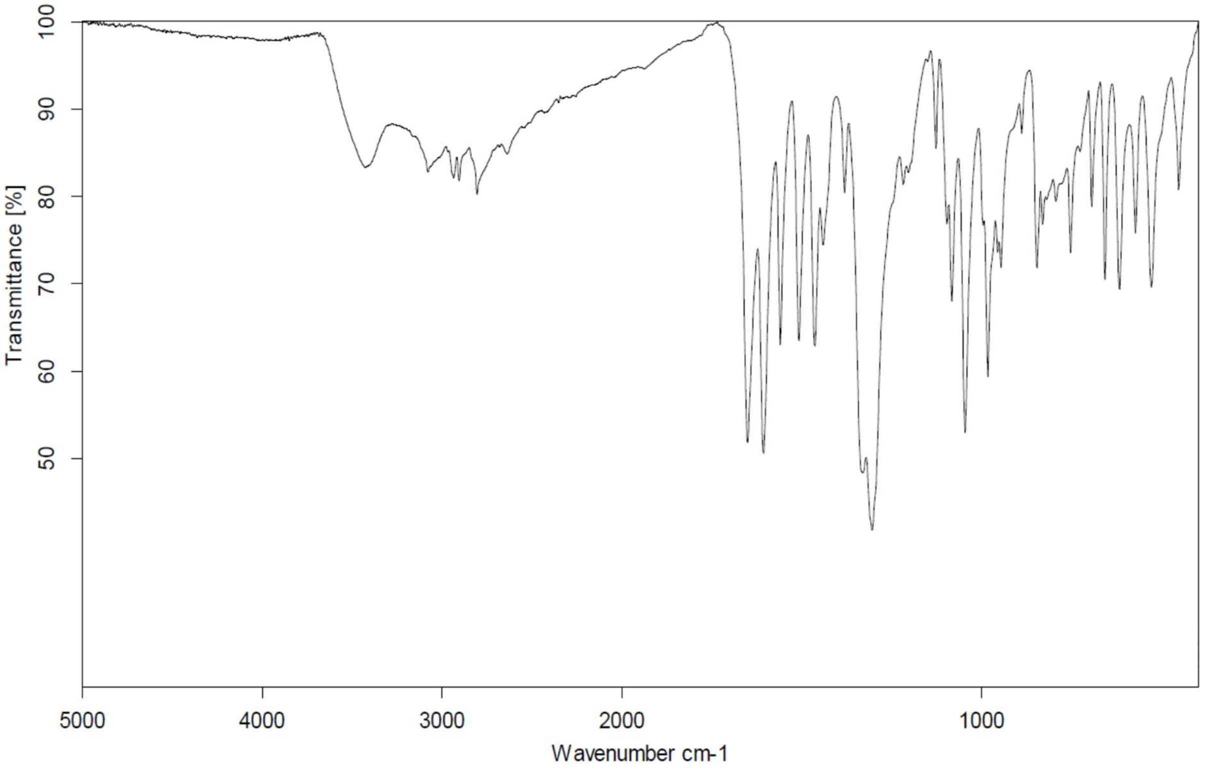

Transmittance [%]
100
90
80
70
60
50
5000
4000
3000
2000
1000
Wavenumber cm-1

Figure S2. The UV-Vis spectra of **[(+)VOL[4]]** and **[(-)VOL[4]]** complexes.

[(+)VOL[4]]

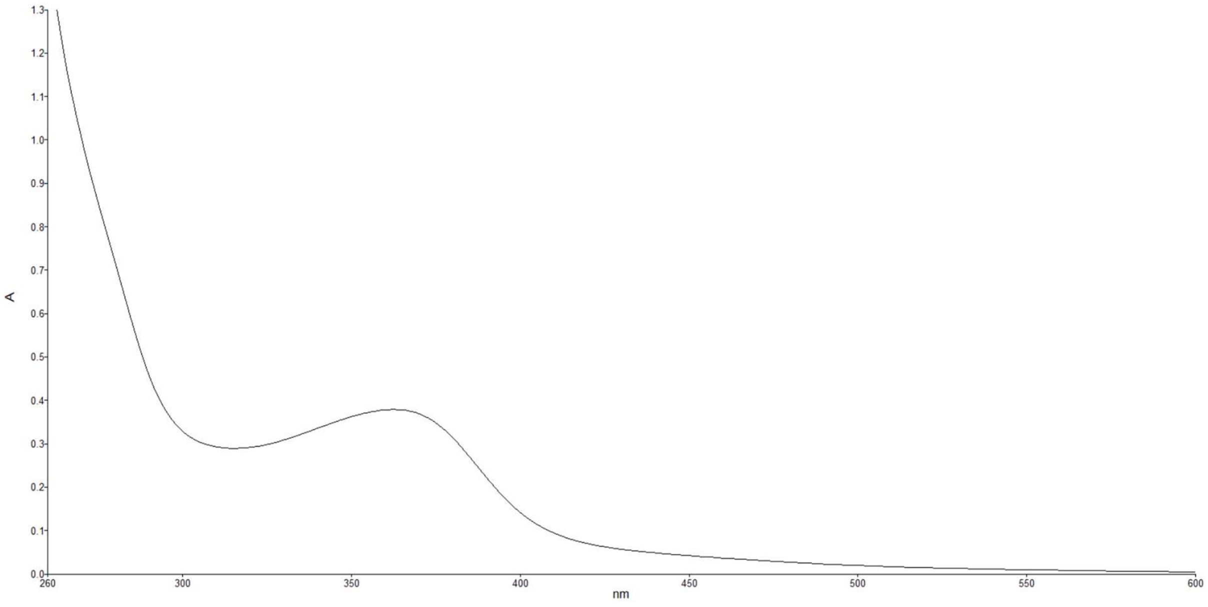

[(-)VOL[4]]

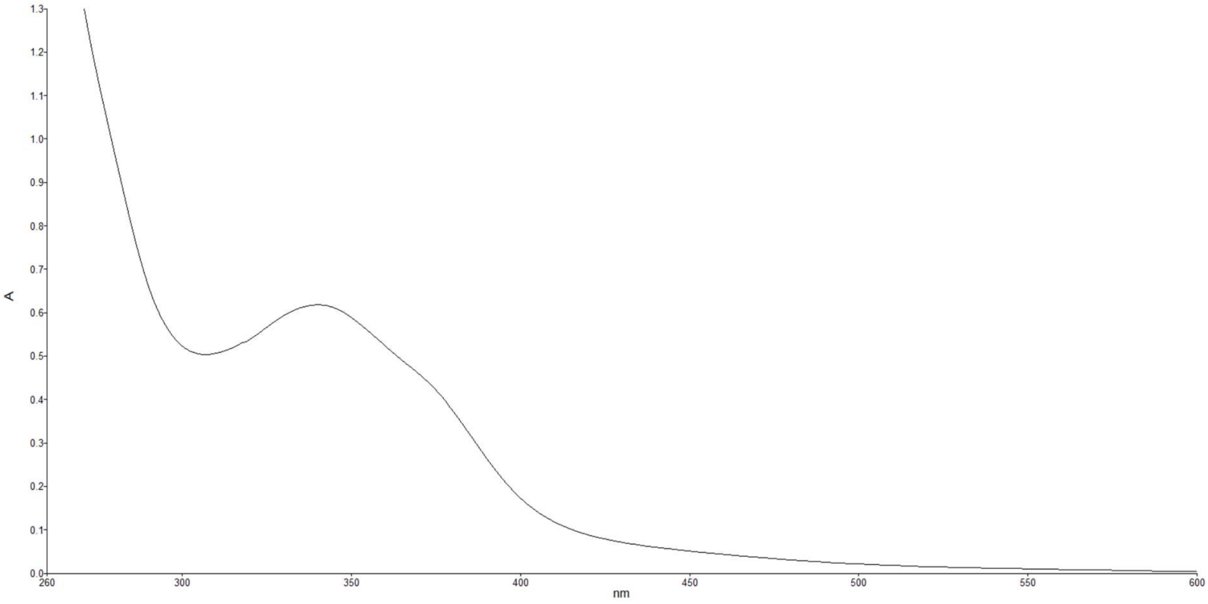

Figure S3. The CD spectra of **[(+)VOL^{4}]** and **[(-)VOL^{4}]** complexes.

[(+)VOL^{4}]

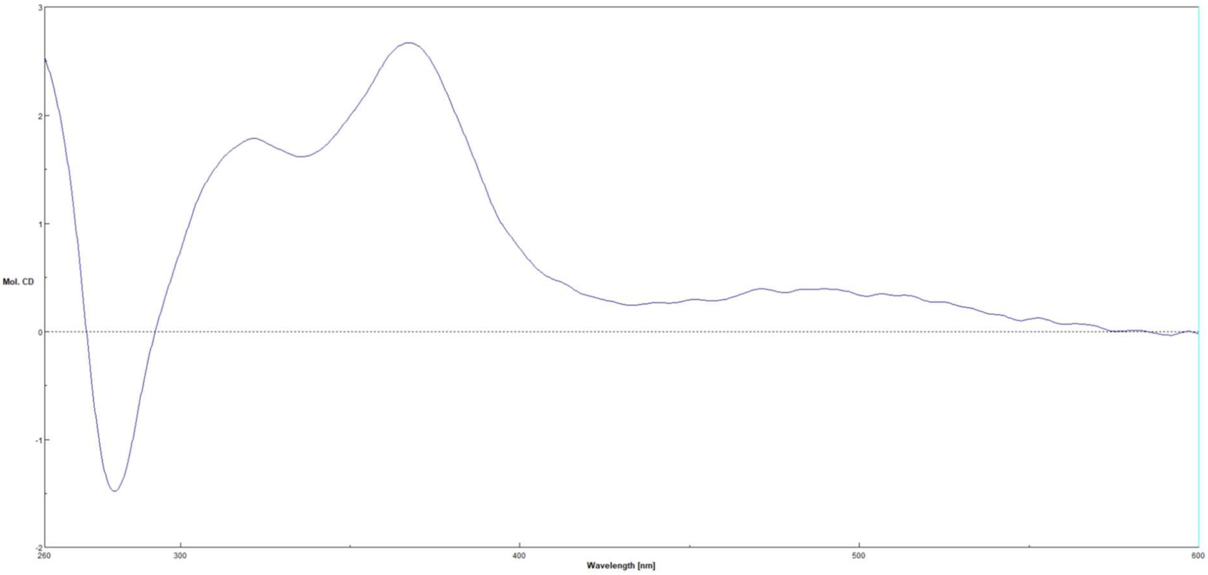

[(-)VOL^{4}]

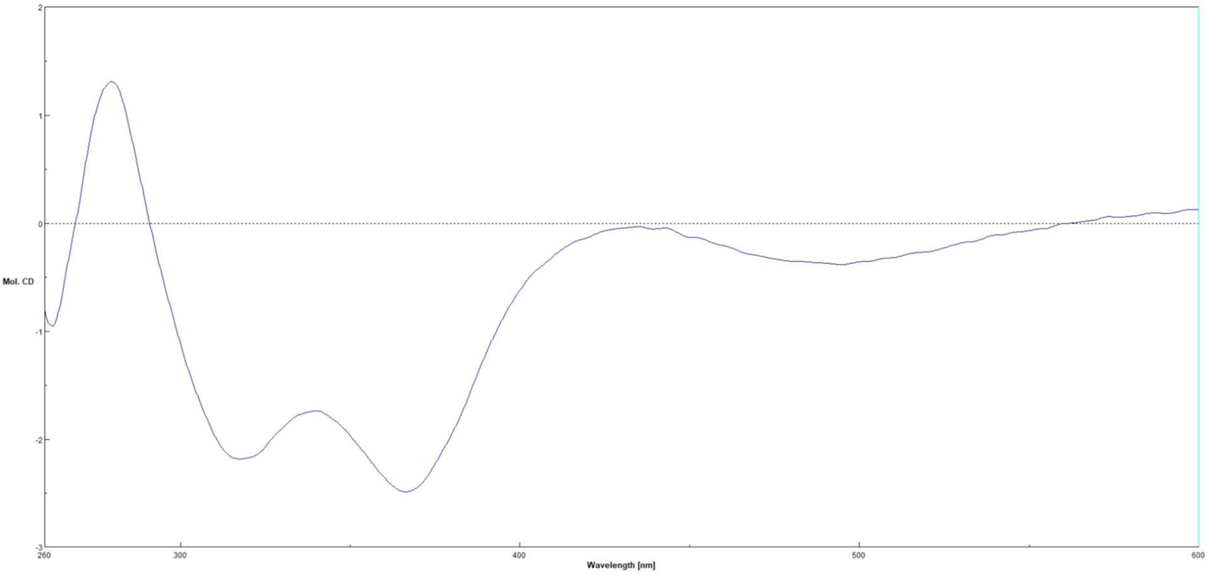

Figure S4. a) ^{1}H, ^{51}V, COSY and NOESY NMR spectra of the **[(+)VOL^{4}]** complex.

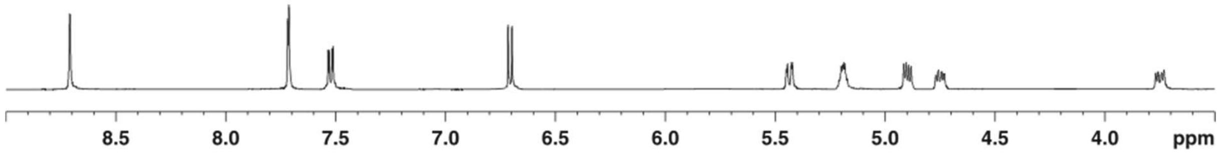

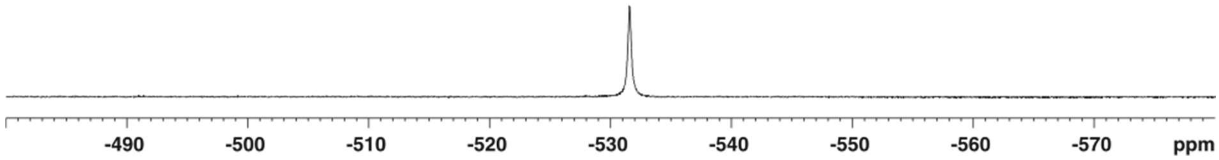

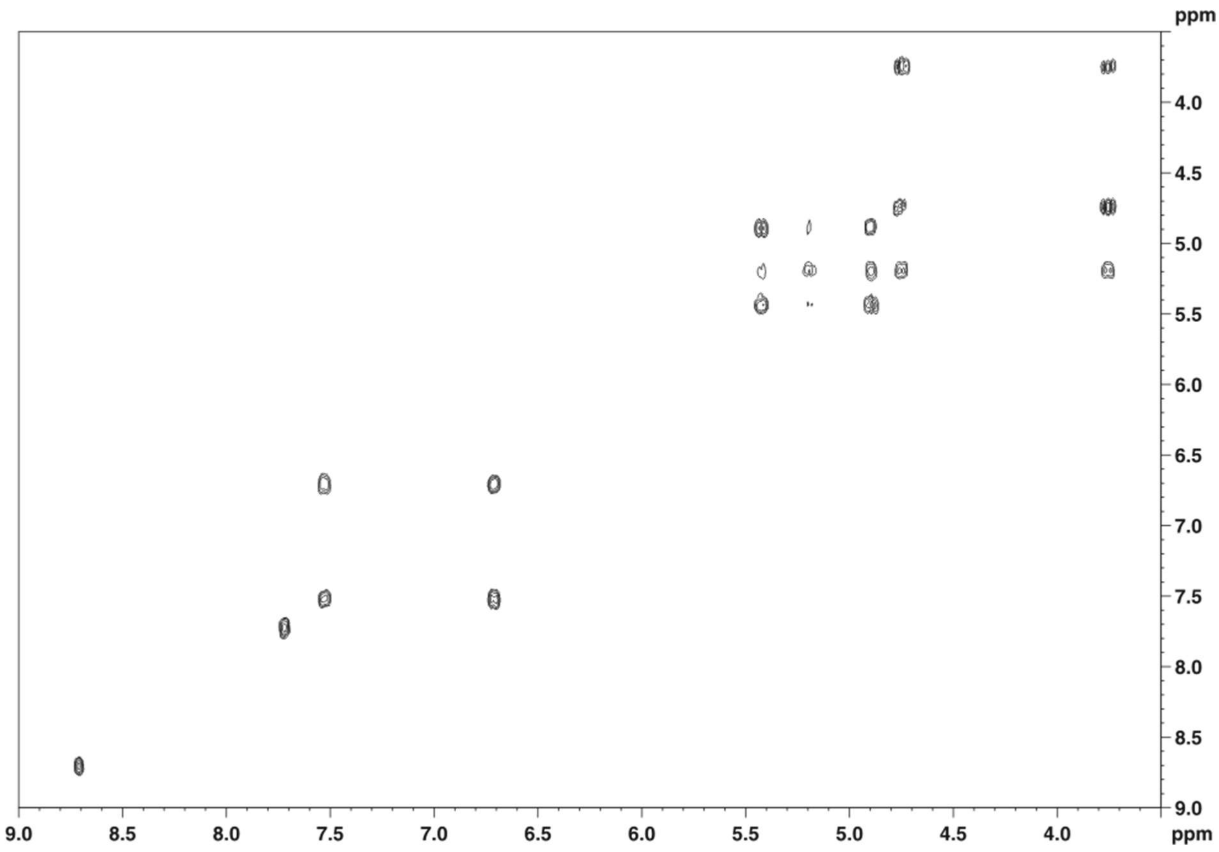

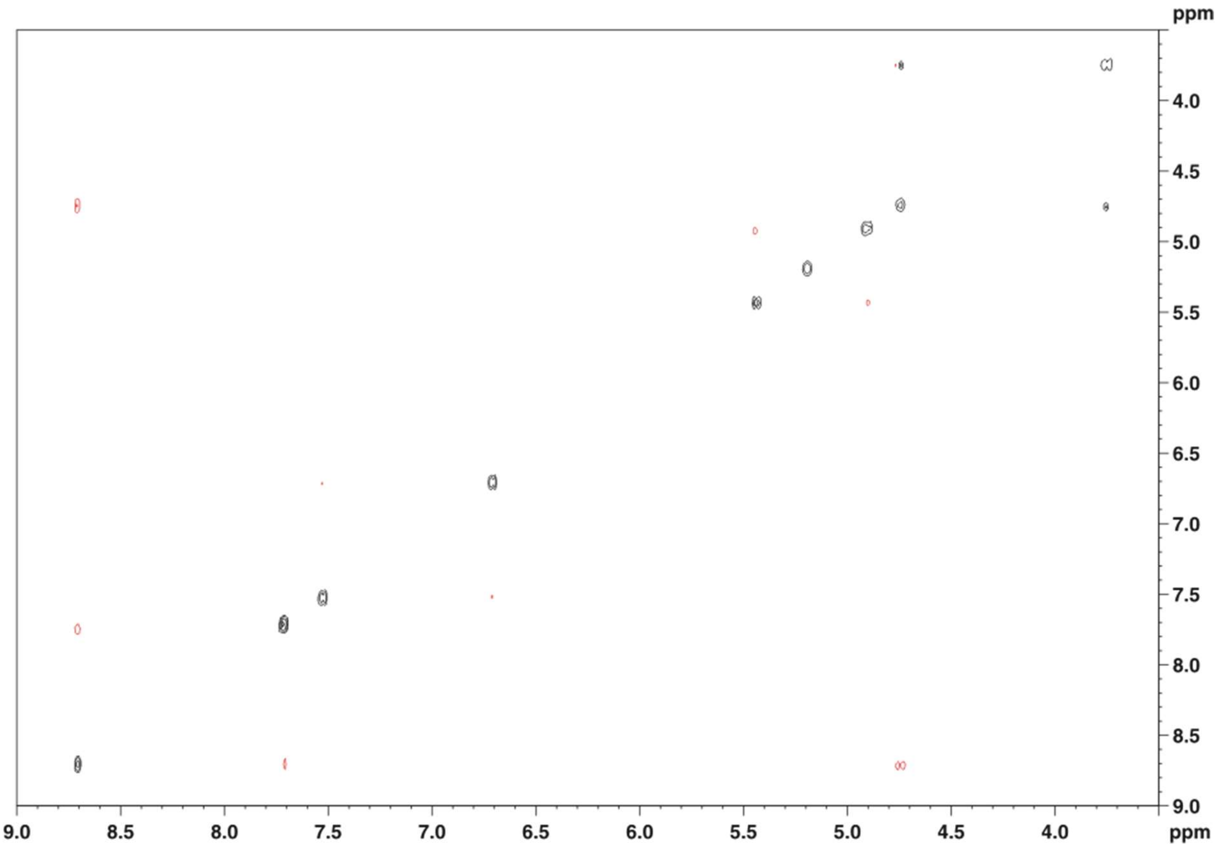

b) ^{1}H and ^{51}V NMR spectra of the **[(+)VOL^{1}], [(+)VOL^{2}]**, **[(+)VOL^{3}]**, **[(+)VOL^{5}]** complexes.

[(+)VOL^{1}]

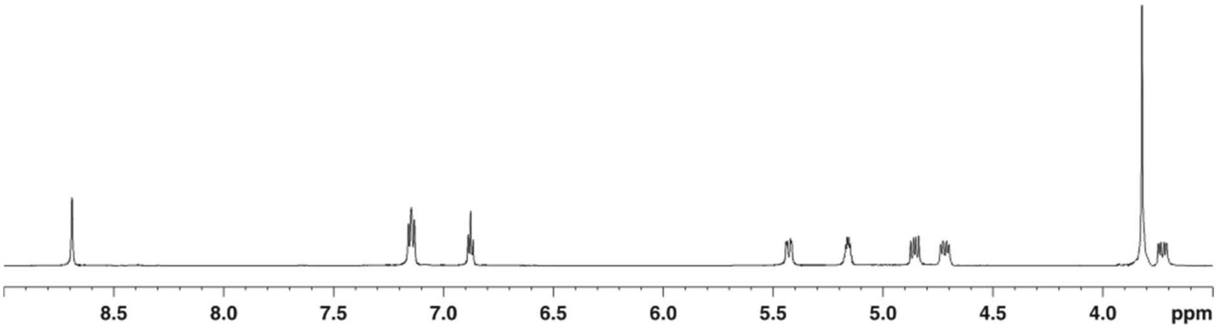

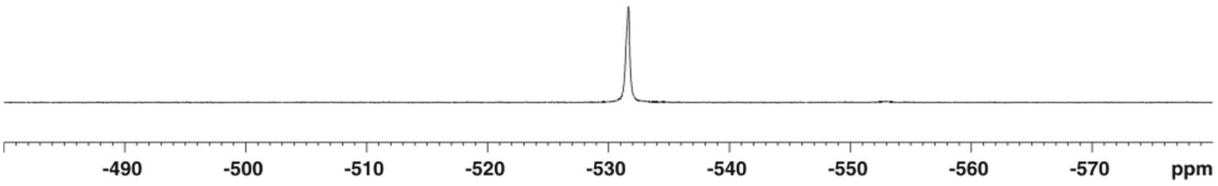

[(+)VOL^2]

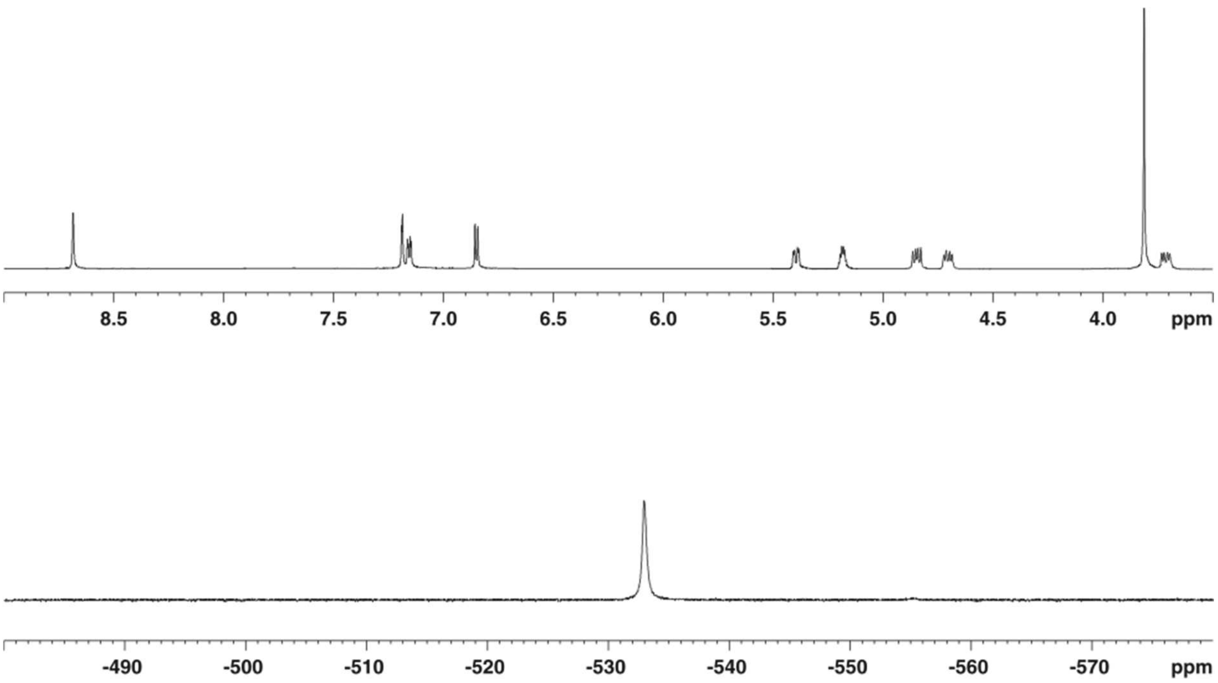
8.5
8.0
7.5
7.0
6.5
6.0
5.5
5.0
4.5
4.0
ppm
-490
-500
-510
-520
-530
-540
-550
-560
-570
ppm

[(+)VOL^3]

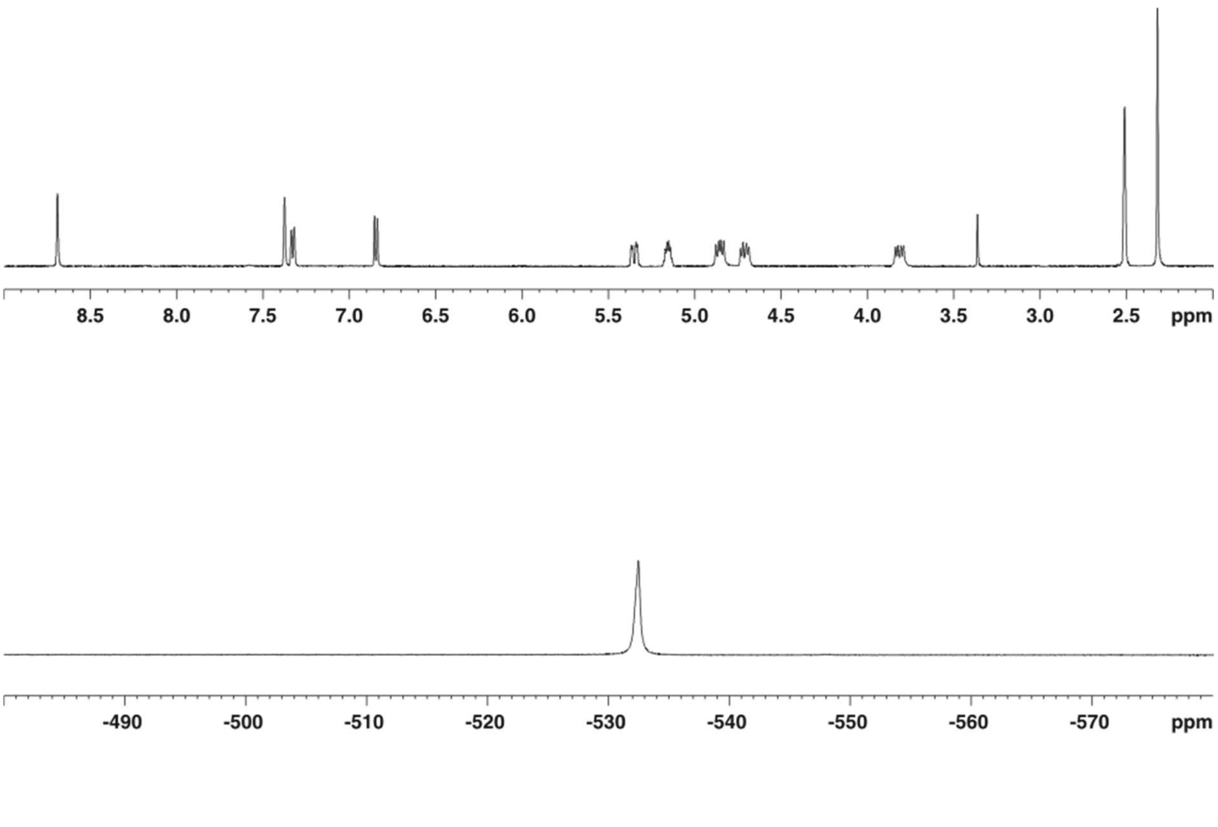
8.5
8.0
7.5
7.0
6.5
6.0
5.5
5.0
4.5
4.0
3.5
3.0
2.5
ppm
-490
-500
-510
-520
-530
-540
-550
-560
-570
ppm

[(+)VOL[5]]

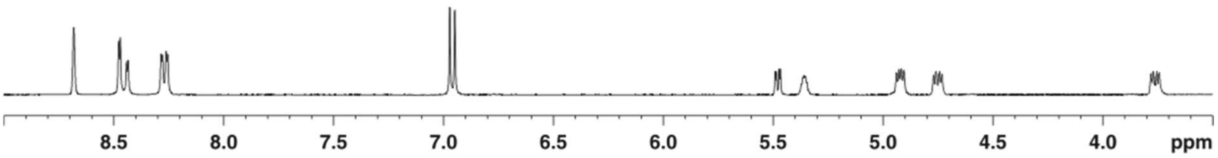
8.5
8.0
7.5
7.0
6.5
6.0
5.5
5.0
4.5
4.0
ppm

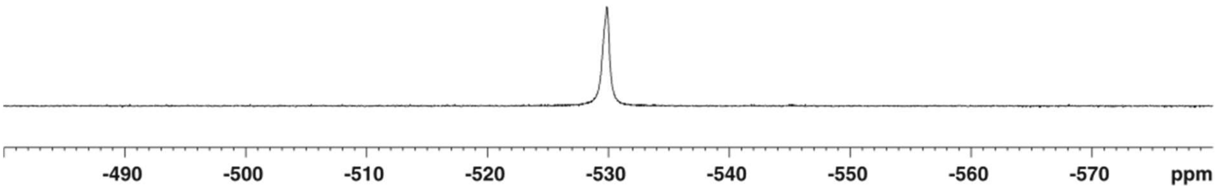
-490
-500
-510
-520
-530
-540
-550
-560
-570
ppm

Figure S5. Images of a) **[(+)VOL^4]** and b) **[(-)VOL^4]** compounds.

a)

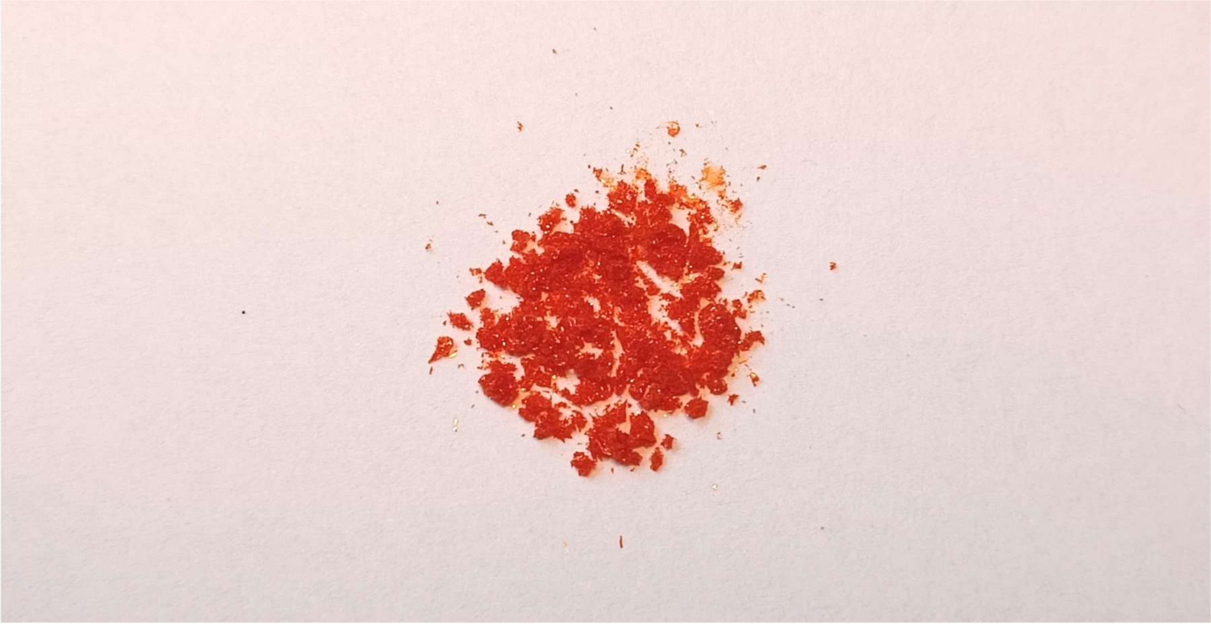

b)

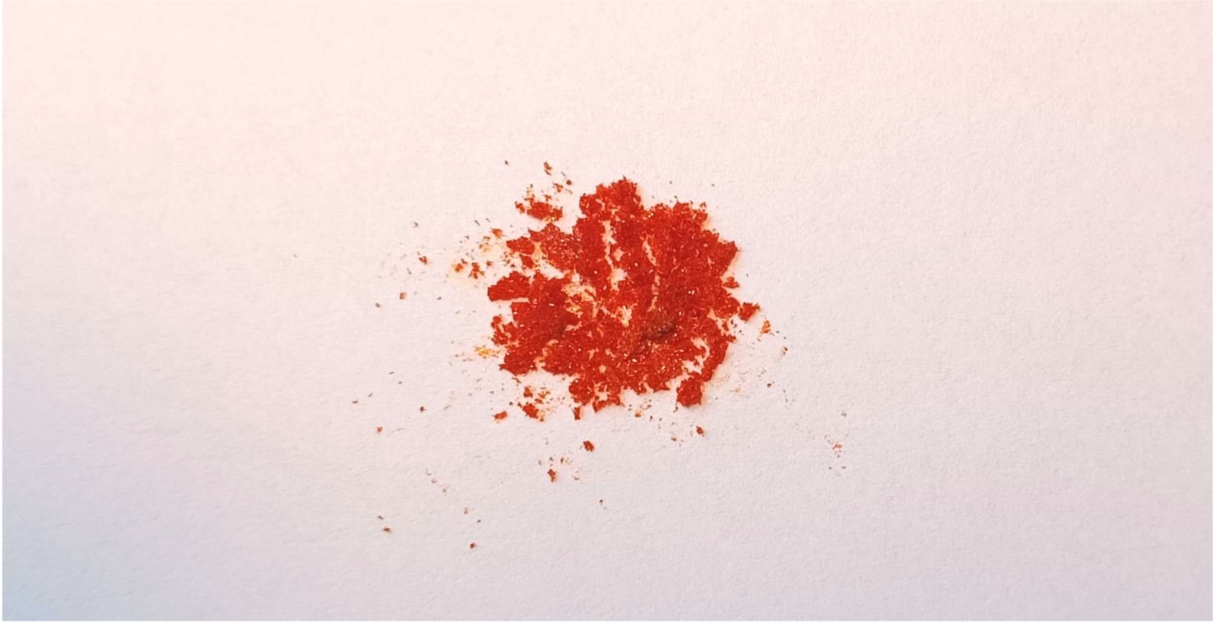

Supplement: Supplementary file 1 [file ijms-25-05010-s001.zip › ijms-2955877-supplementary.pdf]
